# Immunogenicity and efficacy of a novel multi-patch SARS-CoV-2/COVID-19 vaccine candidate

**DOI:** 10.3389/fimmu.2023.1160065

**Published:** 2023-06-19

**Authors:** Beatriz Perdiguero, Laura Marcos-Villar, María López-Bravo, Pedro J. Sánchez-Cordón, Carmen Zamora, José Ramón Valverde, Carlos Óscar S. Sorzano, Laura Sin, Enrique Álvarez, Manuel Ramos, Margarita Del Val, Mariano Esteban, Carmen Elena Gómez

**Affiliations:** ^1^Department of Molecular and Cellular Biology, Centro Nacional de Biotecnología, Consejo Superior de Investigaciones Científicas, Madrid, Spain; ^2^Centro de Investigación Biomédica en Red de Enfermedades Infecciosas (CIBERINFEC), Instituto de Salud Carlos III (ISCIII), Madrid, Spain; ^3^Department of Microbial Biotechnology, Centro Nacional de Biotecnología, Consejo Superior de Investigaciones Científicas, Madrid, Spain; ^4^Veterinary Pathology Department, Centro de Investigación en Sanidad Animal, Instituto Nacional de Investigación y Tecnología Agraria y Alimentaria, Consejo Superior de Investigaciones Científicas, Madrid, Spain; ^5^Scientific Computing, Centro Nacional de Biotecnología, Consejo Superior de Investigaciones Científicas, Madrid, Spain; ^6^Biocomputing Unit and Computational Genomics, Centro Nacional de Biotecnología, Consejo Superior de Investigaciones Científicas, Madrid, Spain; ^7^Centro de Biología Molecular Severo Ochoa, Consejo Superior de Investigaciones Científicas and Universidad Autónoma de Madrid, Madrid, Spain

**Keywords:** SARS-CoV-2, multi-patch vaccine, poxvirus MVA, mice studies, B and T cell immune responses, binding and neutralizing antibodies, efficacy

## Abstract

**Introduction:**

While there has been considerable progress in the development of vaccines against SARS-CoV-2, largely based on the S (spike) protein of the virus, less progress has been made with vaccines delivering different viral antigens with cross-reactive potential.

**Methods:**

In an effort to develop an immunogen with the capacity to induce broad antigen presentation, we have designed a multi-patch synthetic candidate containing dominant and persistent B cell epitopes from conserved regions of SARS-CoV-2 structural proteins associated with long-term immunity, termed CoV2-BMEP. Here we describe the characterization, immunogenicity and efficacy of CoV2-BMEP using two delivery platforms: nucleic acid DNA and attenuated modified vaccinia virus Ankara (MVA).

**Results:**

In cultured cells, both vectors produced a main protein of about 37 kDa as well as heterogeneous proteins with size ranging between 25-37 kDa. In C57BL/6 mice, both homologous and heterologous prime/boost combination of vectors induced the activation of SARS-CoV-2-specific CD4 and CD8 T cell responses, with a more balanced CD8^+^ T cell response detected in lungs. The homologous MVA/MVA immunization regimen elicited the highest specific CD8^+^ T cell responses in spleen and detectable binding antibodies (bAbs) to S and N antigens of SARS-CoV-2. In SARS-CoV-2 susceptible k18-hACE2 Tg mice, two doses of MVA-CoV2-BMEP elicited S- and N-specific bAbs as well as cross-neutralizing antibodies against different variants of concern (VoC). After SARS-CoV-2 challenge, all animals in the control unvaccinated group succumbed to the infection while vaccinated animals with high titers of neutralizing antibodies were fully protected against mortality, correlating with a reduction of virus infection in the lungs and inhibition of the cytokine storm.

**Discussion:**

These findings revealed a novel immunogen with the capacity to control SARS-CoV-2 infection, using a broader antigen presentation mechanism than the approved vaccines based solely on the S antigen.

## 1 Introduction

The coronavirus disease 2019 (COVID-19) pandemic caused by severe acute respiratory syndrome coronavirus 2 (SARS-CoV-2) is causing a major health problem as well as economic and social burden with unprecedented consequences. As of May 2023, more than 767 million cases and over 6.9 million deaths have been reported worldwide and the numbers continue to rise despite the containment measures and the implemented mass vaccination campaigns (https://covid19.who.int). Most of the approved COVID-19 vaccines currently in use target the surface-exposed spike (S) glycoprotein of the original Wuhan-Hu-1 SARS-CoV-2 isolate to induce neutralizing antibodies (NAbs) and have significantly contributed to limit the mortality associated with the virus infection. However, the emergence of new viral variants with high mutation rates in the S protein and the virus enhanced transmissibility have reduced the efficacy of most of the vaccines and therapies based on NAbs, in particular in vulnerable populations (1, 2). In addition, natural infection and vaccination induced short-lived binding and neutralizing antibodies and, hence, a third or even a fourth vaccine dose is required to boost humoral immunity and to protect against newly emerging variants (3–5). To overcome this inconvenience, it is desirable the design of next-generation vaccines that could provide broad coverage and long-term protection, in order to avoid or contain ongoing viral evolution and future emerging outbreaks.

Immunological studies in recovered COVID-19 patients revealed that a combination of SARS-CoV-2-specific antibodies, memory B cells and CD4 and CD8 T cells are likely to be beneficial in minimizing COVID-19 severity and to achieve clinical short-term protection (6, 7). However, it is early to know how long these responses are maintained, as mass vaccination started in 2021. Follow-up studies from patients who recovered from the closely related coronavirus SARS-CoV-1 outbreak revealed that virus-specific humoral responses significantly decline 1 year post-infection while long-lasting memory CD4 and CD8 T cells could be detected as long as 11 years after infection (8). The lack of specific memory B cell response in SARS-CoV-1 survivors 6 years after disease onset (9) reinforces the potential role that virus-specific memory T cells could have in long-term protection against coronavirus infection and, thus, next-generation vaccines also need to confer long-term activation of this arm of the immune response.

In the context of B cell responses, the greatest number of epitopes recognized by sera from COVID-19 convalescents are derived from the spike glycoprotein (S), nucleoprotein (N) and membrane protein (M) (10), being the receptor binding domain (RBD) region of the S protein the major target of NAbs (11, 12). However, RBD mutations introduced in the emerging virus variants as result of the immune pressure exerted by infection or vaccination have significantly impacted the efficacy of the humoral response targeting this domain (13).

The aim of the present study is the design and preclinical evaluation of a novel polyvalent multi-patch immunogen, termed CoV2-BMEP (B cell Multiepitopic Protein), containing dominant and persistent B cell epitopes from conserved regions of SARS-CoV-2 structural proteins when expressed by either DNA or MVA vectors. In addition to the B cell epitopes, the selected regions include embedded HLA class I and II sequences for induction of both CD8 and CD4 T cell responses. Although the RBD region from the S protein is the main target of NAbs, we decided to exclude this region from the design due to the high number of aminoacid mutations that accumulate during the virus evolution. Hence, most of the S protein sequences included in CoV2-BMEP (6 out of 8) are from the conserved S2 domain. The highly conserved fusion peptide (FP) sequence has been included since it represents a cryptic epitope target by broadly NAbs.

## 2 Materials and methods

### 2.1 Design of chimeric CoV2-BMEP polyvalent multi-patch immunogen targeting B cells

**Figure 1:**
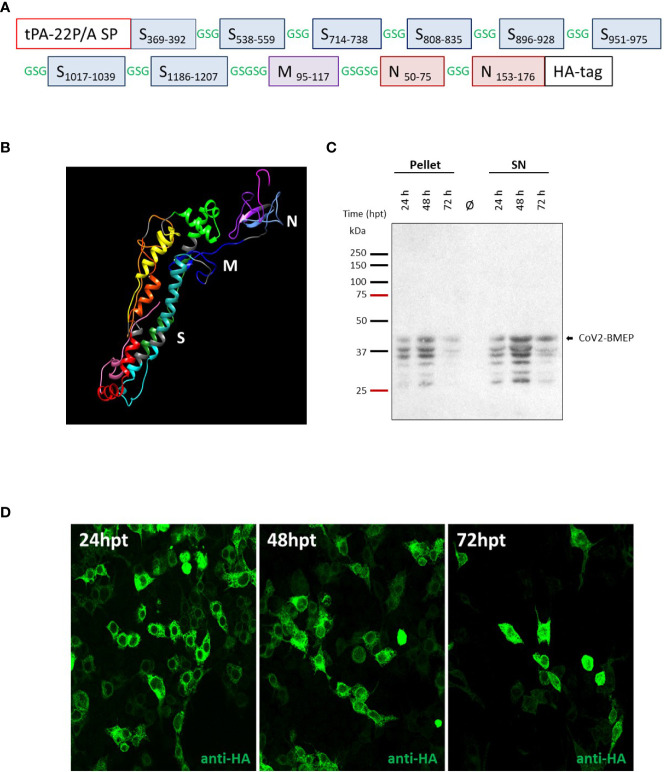
*In vitro* characterization of the DNA vector expressing the polyvalent multi-patch CoV2-BMEP protein. **(A)** Schematic representation of the chimeric polyvalent multi-patch vaccine CoV2-BMEP targeting B cells. **(B)** 3D structure prediction of the CoV2-BMEP protein. **(C, D)** Time-course expression of CoV2-BMEP protein after DNA transfection by western-blotting and confocal microscopy analyses. **(C)** Transfected HEK-293T cells were harvested at 24, 48 and 72 hours post-transfection and pellets and supernatants (SN) were processed as described in Materials and Methods, fractionated by 12% SDS-PAGE and analyzed by western-blotting using the rabbit polyclonal anti-HA antibody. **(D)** Subcellular localization of CoV2-BMEP protein by confocal microscopy. Transfected HeLa cells were fixed at 24, 48 and 72 hours post-transfection and labeled with a rabbit polyclonal anti-HA antibody. After incubation with the corresponding secondary antibody conjugated with Alexa Fluor 488 (green staining), cells were stained with DAPI to detect cell nuclei and visualized by confocal microscopy.

The selection of viral fragments comprising the multi-patch CoV2-BMEP vaccine candidate was based on the following criteria: (i) inclusion of major dominant and persistent antigenic domains in the SARS-CoV-2 proteome with high conservation rates across human endemic coronaviruses (HCoVs) and low level of mutations that have been associated with protection in SARS-CoV-1 and SARS-CoV-2 survivors; (ii) presence of beneficial B cell epitopes as well as CD4 and CD8 T cell epitopes restricted by a wide range of HLA class I and II molecules to induce cross-protective responses with high global population coverage and; (iii) exclusion of decoy epitopes irrelevant for viral control that might compromise the safety of the vaccines. Viral patches included in the CoV2-BMEP synthetic protein were selected by manual sequence inspection of immunodominant regions or B and T cell epitopes recurrently recognized by SARS-CoV and SARS-CoV-2 survivors during natural infection or vaccination published in research articles (10, 14–37). The S, M and N protein sequences of six known human coronavirus strains, including SARS-CoV-2 (NC_045512), SARS-CoV (NC_0041718), MERS-CoV (NC_019843), HCoV-229E (NC_002645), HCoV-OC43 (NC_006213), HCoV-NL63 (NC_005831) and HCoV-HKU1 (NC_002645) were downloaded from the NCBI (National Center for Biotechnology Information). Sequence alignment was performed using ClustalW (version 2.0.12) with default parameters to determine the major blocks of similarity. Reported common epitopes that consistently remain reactive in more than 80% of COVID-19 samples up to 180-220 days post-symptom onset were defined by Li et al. (38) as dominant and persistent epitopes. CoV2-BMEP includes 11 antigenic patches: 8 from S protein (2 from S1 and 6 from S2 domains), 1 from M protein and 2 from N protein (**Supplementary Table 1**) that were connected using the flexible GSGSG linker and ordered following the SARS-CoV-2 genomic organization (**Figure 1A**). A mutant form of the signal sequence of the human tissue plasminogen activator (tPA-22P/A SP) was added at the N-terminus, which has been reported to significantly enhance the secretion of heterologous antigens (39), whereas at the C-terminus the HA-tag was inserted for expression analysis.

### 2.2 *In silico* characterization of CoV2-BMEP synthetic protein

ExPASy ProtParam online service (https://web.expasy.org/protparam/) was used to predict physico-chemical features of the multi-patch vaccine candidate. The parameters include the average molecular weight (MW), the number of amino acid residues, the theoretical isoelectric point (pI), extinction coefficients, estimated *in vitro* and *in vivo* half-life, aliphatic index, instability index and grand average of hydropathicity (GRAVY). The antigenicity of CoV2-BMEP was predicted using ANTIGENpro bioinformatics tool (http://scratch.proteomics.ics.uci.edu/), which is based on pathogen-independent, sequence-based, alignment-free analysis (40). For allergenicity prediction, AllergenFP server (http://www.ddgpharmfac.net/AllergenFP/) was used (41).

### 2.3 Structure prediction of chimeric CoV2-BMEP multi-patch protein

Since the designed vaccine was composed of multiple epitopes from diverse origins joined by linker sequences, homology-based predictive methods were considered unlikely to succeed. For this reason, homology-based methods were complemented with a variety of *ab initio* prediction methods using either public web-server or in-house deployments. The putative structure of the synthetic vaccine sequence was predicted using CABSfold (42), C-QUARK (43), IntFold (44), I-TASSER (45), Raptor-X (46), Sparks (47) and trRosetta (48). Additional partial models using CABS, DNCON2 (49) and UNICON3D (50)/NNCON (51) were generated to further complement predicted model. Since AlphaFold (52, 53) was not publicly available at the time, it was not used in the original predictions, but it has been included afterwards to compare its results. The resulting predictions were assorted manually, comparing predicted domains with their native structure and inspecting relative arrangement and commonality among predicted models. As we were interested in epitope exposition, a worst-case scenario was also considered identifying models with maximal packing and minimal exposition.

### 2.4 Immunoinformatic analysis

The sequence was subjected to a confirmatory prediction of antigenic sites using the imed.med.ucm.es server (http://imed.med.ucm.es), the epitope prediction and the Immune Epitope Database (IEDB) analysis tools from immuneepitope.org (54) and tepitool (55) for human MHC classes I and II (DP, DQ and DR) binding and worldwide population coverage prediction by areas using data from the IEDB Database as of 2020-08-11, with the recommended IEDB methods. The predicted coverage for MHC classes I and II epitopes for CoV2-BMEP construct is detailed in **Supplementary Tables 2** and **3**.

### 2.5 Cells

HEK-293T cells (ATCC catalog no. CRL-3216), HeLa cells (ATCC CCL-2), DF-1 cells (ATCC CRL-12203) and Vero-E6 cells (ATCC CRL-1586) were grown in Dulbecco’s Modified Eagle’s Medium (DMEM; Sigma-Aldrich, St. Louis, MO, USA) supplemented with 100 U/mL penicillin/100 µg/mL streptomycin (Sigma-Aldrich), 2 mM L-glutamine (Sigma-Aldrich), 0.1 mM non-essential amino acids (Sigma-Aldrich), 0.5 μg/mL amphotericin B (Fungizone; Gibco-Life Technologies, Waltham, MA, USA) and 10% heat-inactivated fetal bovine serum (FBS; Sigma-Aldrich) for HEK-293T, DF-1 and Vero-E6 cells or 10% heat-inactivated newborn calf serum (NCS; Sigma-Aldrich) for HeLa cells. Cell lines were maintained in a humidified air, 5% CO_2_ atmosphere at 37°C.

### 2.6 Viruses

The poxviruses used in this work included: the attenuated wild-type modified vaccinia virus Ankara (MVA-WT) derived from the Ankara strain after 586 serial passages in chicken embryo fibroblast (CEF) cells (provided by Prof. Dr. Gerd Sutter, Ludwig-Maximilians-University of Munich, Munich, Germany) and MVA-CoV2-BMEP, in which CoV2-BMEP gene was inserted into the thymidine kinase (TK) locus (*J2R* gene) of the parental MVA-WT virus, and generated in this work. Poxviruses were grown in CEF cells with supplemented DMEM–2% FBS and purified through two 36% (w/v) sucrose cushions. Viral titers were determined by immunostaining plaque assay in DF-1 cells, as previously described (56).

SARS-CoV-2 strain MAD6 (provided by Prof. Dr. Enjuanes and Dr. Honrubia, CNB-CSIC, Madrid, Spain) is a virus isolated from a nasopharyngeal swab of a 69-year-old male COVID-19 patient from Hospital 12 de Octubre in Madrid, Spain. The viral stock was prepared as previously described (57). Virus infectivity titers were determined by median tissue culture infectious dose (TCID_50_) or standard plaque assays in Vero-E6 cells. Full-length virus genome was sequenced, being identical to the SARS-CoV-2 reference sequence (Wuhan-Hu-1 isolate, GenBank: MN908947), except for the silent mutation C3037 > T and two mutations leading to amino acid changes: A23403 > G (in S protein) and C14408 > T (in nsp12). SARS-CoV-2 virus stocks derived from VoCs B.1.1.7 (Alpha; hCoV-19/Belgium/rega-12211513/2020; EPI_ISL_791333, 2020-12-21), B.1.351 (Beta; hCoV-19/Belgium/rega-1920/2021; EPI_ISL_896474, 2021-01-11), B.1.167.2 (Delta; hCoV-19/Belgium/rega-7214/2021, EPI_ISL_2425097) and B.1.1.529 (Omicron; hCoV-19/Belgium/rega-20174/2021, EPI_ISL_6794907) (Institut Pasteur, Paris, France) were isolated from Belgian patients and characterized by next-generation sequencing.

### 2.7 DNA vectors

CoV2-BMEP construct was synthesized and subcloned into the pcDNA vector backbone by GeneArt (Thermo Fisher Scientific, Waltham, MA, USA), resulting the plasmid pcDNA-CoV2-BMEP (shortly DNA-CoV2-BMEP). The plasmid transfer vector pCyA20-CoV2-BMEP was obtained by Gibson assembly system (New England Biolabs, Ipswich, MA, USA) according to manufacturer’s instructions and using pCyA20 (58) and DNA-CoV2-BMEP plasmids as templates. The sequence of the resulting plasmid was confirmed by PCR and DNA sequence analysis (Macrogen, Seoul, South Korea). The pCyA20-CoV2-BMEP (8579 bp) plasmid was used for the generation of MVA-CoV2-BMEP recombinant virus allowing the insertion of CoV2-BMEP construct into the viral TK locus of MVA-WT.

### 2.8 Time-course expression of CoV2-BMEP construct expressed from DNA vector

To determine the correct expression and subcellular localization of CoV2-BMEP construct, monolayers of HEK-293T cells grown in 12-well plates or HeLa cells grown in 24-well plates on glass coverslips were transfected with 1 μg (12-well plates) or 0.5 μg (24-well plates) of DNA-ϕ or DNA-CoV2-BMEP using Lipofectamine-2000 (Invitrogen, Carlsbad, CA, USA) according to manufacturer’s recommendations. At 24, 48 and 72 hours post-transfection, cells were harvested and processed for western-blotting (HEK-293T) or confocal microscopy (HeLa) analyses.

For western-blotting analysis, HEK-293T-transfected cells were collected and cellular pellets and supernatants were obtained as previously described (59), fractionated by 12% Sodium Dodecyl Sulfate-Polyacrylamide Gel Electrophoresis (SDS-PAGE) and analyzed using the rabbit polyclonal anti-HA antibody (1:10,000; Sigma-Aldrich) to evaluate the expression of CoV2-BMEP. Horseradish peroxidase (HRP)-conjugated anti-rabbit (1:5,000) antibody (Sigma-Aldrich) was used as secondary antibody. The immunocomplexes were detected using an enhanced-chemiluminescence system (ECL Plus; GE Healthcare, Chicago, IL, USA).

For confocal microscopy analysis, HeLa-transfected cells were washed with phosphate buffered saline (PBS) and fixed with 4% paraformaldehyde (PFA) in PBS for 15 min. at room temperature (RT). Coverslips were then washed twice with PBS and non-specific unions were blocked with PBS 1X - 4% bovine serum albumin (BSA) at RT for 30 min. After one wash with PBS, cells were permeabilized with PBS 1X - 0.25% saponin, washed once with PBS and incubated with a rabbit polyclonal anti-HA antibody (1:200; Sigma-Aldrich) over night at 4°C. Next day, coverslips were washed 3 times with PBS and incubated with goat anti-rabbit-Alexa Fluor 488 secondary antibody (green staining; diluted 1:500; Life Technologies, Carlsbad, CA, USA) in the dark for 1h at RT. Cells were then washed 3 times with PBS and incubated with 4’,6-diamidino-2-phenylindole (DAPI; 1:200; Sigma-Aldrich) at RT for 20 min. to stain cell nuclei. After, coverslips were washed again with PBS and placed on glass slides using ProLong Gold anti-fade reagent (Invitrogen). Finally, a Leica TCS SP5 microscope was used for the acquisition of optical sections of the cells and the specialized software LasAF (Leica Microsystems) was used for image recording and processing. Image co-localization analysis was performed using LasX software with the co-localization license.

### 2.9 Construction of MVA-CoV2-BMEP recombinant virus

The generation of the MVA-CoV2-BMEP recombinant virus was performed by homologous recombination as previously reported (60). Briefly, 3 × 10^6^ DF-1 cells were infected with MVA-WT at 0.1 plaque-forming units (PFU)/cell and transfected 1 h later with 6 µg of pCyA20-CoV2-BMEP using Lipofectamine-2000 (Invitrogen) and following the manufacturer’s instructions. At 48 h post-infection (h.p.i.), cells were collected, lysed by freeze-thaw cycling, sonicated and used for the screening of MVA recombinants. During the first four plaque purification steps, MVA recombinant viruses transiently co-expressing β-galactosidase (β-Gal; *lacZ* marker gene) and containing the *CoV2-BMEP* gene were isolated from DF-1 cells grown in 6-well plates and stained with 5-bromo-4-chloro-3-indolyl β-D-galactopyranoside (X-Gal; 1.2 mg/mL; Sigma-Aldrich). In the following two isolation steps, MVA recombinants having deleted *lacZ* gene and containing *CoV2-BMEP* gene were isolated by screening for non-stained viral plaques in DF-1 cell monolayers in the presence of X-Gal. The resulting MVA-CoV2-BMEP recombinant was grown in DF-1 cells and the viral crude preparation obtained (P2 stock) was used to expand the virus in large cultures of CEF cells, followed by a purification step through two 36% (w/v) sucrose cushions (P3 stock). Viral titers were determined by immunostaining plaque assay in DF-1 cells, as previously described (56). The viral stocks were free of mycoplasma, bacteria and fungi contamination.

### 2.10 Characterization of MVA-CoV2-BMEP recombinant virus

#### 2.10.1 PCR analysis of viral tk locus

To define the identity and purity of MVA-CoV2-BMEP viral preparation, DNA was extracted from DF-1 cells infected with MVA-WT or MVA-CoV2-BMEP at 5 PFU/cell for 24 h as previously described (61) and used as template for the analysis of the TK locus by PCR. The amplification reactions were performed using the Phusion High-Fidelity DNA polymerase (BioLabs, Ipswich, MA, USA), according to the manufacturer’s recommendations, and the primers TK-L: 5′-TGATTAGTTTGATGCGATTC-3′ and TK-R: 5′-CTGCCGTATCAAGGACA-3′ spanning TK flanking regions.

#### 2.10.2 Time-course expression of cov2-bmep protein by western-blotting analysis

To determine the correct expression of CoV2-BMEP protein from MVA-CoV2-BMEP virus, HeLa cells grown in 24-well plates were mock-infected or infected with MVA-CoV2-BMEP at 3 PFU/cell. At 0, 6, 16 and 24 h.p.i., cellular pellets and supernatants were obtained, fractionated by 12% SDS-PAGE, and analyzed by western-blotting using the rabbit polyclonal anti-HA antibody for the detection of CoV2-BMEP protein. The rabbit polyclonal anti-actin antibody (1:1,000; Sigma-Aldrich) was used as loading control.

#### 2.10.3 Subcellular localization of CoV2-BMEP protein by confocal microscopy analysis

To evaluate the subcellular localization of CoV2-BMEP protein expressed from MVA-CoV2-BMEP virus, HeLa cells grown in 24-well plates on glass coverslips were infected with MVA-CoV2-BMEP at 1 PFU/cell. At 16 h.p.i., infected cells were stained with wheat germ agglutinin (WGA) probe conjugated to Alexa Fluor 555 (red staining; diluted 1:500; Invitrogen) for 2 min. at RT and fixed with 4% PFA for 15 min. at RT. After, coverslips were treated as described above for DNA-CoV2-BMEP-transfected cells. In addition to a rabbit polyclonal anti-HA antibody (1:200; Sigma-Aldrich), a rabbit polyclonal antibody against SARS-CoV-2 N protein (1:200; Sino Biological, Beijing, P.R. China) was used.

#### 2.10.4 Genetic stability of MVA-CoV2-BMEP

The stability of CoV2-BMEP protein expressed from MVA-CoV2-BMEP virus was analyzed after serial infection passages in DF-1 cells grown in T25 tissue culture flasks as previously described (62). The correct expression of CoV2-BMEP protein in 22 isolated plaques from stability passage 7 was determined by western-blotting analysis using the rabbit polyclonal anti-HA antibody (Sigma-Aldrich), followed by goat anti-rabbit-HRP (Sigma-Aldrich). The immunocomplexes were detected using an ECL system (GE Healthcare).

#### 2.10.5 CoV2-BMEP recognition by CD8 T lymphocytes from SARS-CoV-2 convalescents

Frozen PBMCs from 10 SARS-CoV-2-infected and fully recovered volunteers from CSIC staff were thawed in RPMI+10% human AB serum. A fraction of PBMCs was infected 24 h later with MVA-WT or MVA-CoV2-BMEP viruses at a moi of 5, incubated for 1 h at 37°C for virus absorption, washed and incubated in RPMI+10% FCS for 5 h of infection. After this time, an equal number of PBMCs from the same patient was added as responder cells and incubated for a further 2 h. Thereafter, ER/Golgi transport inhibitors (monensin and brefeldin A) were added to allow the accumulation of intracellular cytokines and incubated overnight at 37°C. Next day, cells were surface stained with APC–anti-CD8α, fixed and incubated with PE–anti–IFN-γ or Alexa Fluor 488-anti-CD107a during permeabilization (Dako; (63)) according to conventional procedures. Events were acquired in a BD FACSCanto II flow cytometer and data analysed using BD FACSDiva software.

### 2.11 Mouse immunizations

#### 2.11.1 Effect of CoV2-BMEP expression on immune cell recruitment

To evaluate the effect of CoV2-BMEP expression from DNA or MVA vectors on immune cell recruitment in muscle and draining lymph nodes (DLNs), two different *in vivo* assays were performed. For the analysis of the effect of CoV2-BMEP expression from DNA vector, two groups of female 6-8*-*week*-*old C57BL/6JOlaHsd mice (*n* = 9), pursached from Envigo Laboratories, were immunized with 25 μg of DNA-CoV2-BMEP or DNA-φ by the intramuscular (i.m.) route (groups 1 and 2). PBS-treated animals were used as the control group (group 3). At days 1, 3 and 7 post-inoculation, 3 animals of each group were sacrificed and total muscle from the site of inoculation and DLNs were harvested and processed for the analysis by flow cytometry of different immune cell populations. To evaluate the effect of CoV2-BMEP expression from MVA vector, two groups of female C57BL/6JOlaHsd mice (*n* = 4), were immunized with 1 x 10^7^ PFU of MVA-WT or MVA-CoV2-BMEP by the i.m. route (groups 1 and 2). PBS-treated animals were used as the control group (group 3). At day 1 post-inoculation, animals were sacrificed and muscle from the site of inoculation and DLNs were collected and processed for the evaluation by flow cytometry of different immune cell populations.

#### 2.11.2 SARS-CoV-2-Specific immunogenicity elicited by CoV2-BMEP protein

To characterize the SARS-CoV-2-specific immunogenicity elicited by CoV2-BMEP protein expressed from DNA or MVA vectors, five groups of female C57BL/6JOlaHsd mice (*n* = 5) were immunized with 50 μg of DNA-CoV2-BMEP or DNA-φ or with 1 x 10^7^ PFU of MVA-CoV2-BMEP or MVA-WT by the i.m. route. Four weeks later (day 28), mice were immunized with 50 μg of DNA-CoV2-BMEP or with 1 x 10^7^ PFU of MVA-CoV2-BMEP or MVA-WT by the i.m. route in homologous (groups 1, 3 and 5) or heterologous (groups 2 and 4) combinations. At 10 days post-boost (day 38), animals were sacrificed, spleens and lungs were processed for intracellular cytokine staining (ICS) assay and sera were collected for enzyme-linked immunosorbent assay (ELISA) to determine SARS-CoV-2-specific cellular and humoral adaptive immune responses, respectively.

#### 2.11.3 Analysis of MVA-CoV2-BMEP capacity to protect mice against SARS-CoV-2 infection

For the efficacy study, three groups of female 6-8-week-old k18-hACE2 (034860-B6.Cg-Tg(k18-ACE2)2Prlman/J) humanized Tg mice (*n* = 6), purchased from Jackson Laboratory, were immunized by the i.m. route with 1 x 10^7^ PFU of MVA-CoV2-BMEP (group 1) or with PBS (groups 2 and 3) at days 0 and 21. At 4 weeks post-boost (day 47), mice were slightly anesthetized with isoflurane and challenged by the intranasal (i.n.) route with 1 × 10^5^ PFU of SARS-CoV-2 virus (MAD6 isolate) (groups 1 and 2; group 3 remained unchallenged). Animals were then monitored daily for body weight variations and survival for 14 days post-challenge. Mice with more than a 25% of weight loss were sacrificed and lungs and serum samples were harvested. The 4-lobulated right lung was divided longitudinally in two, with one fragment stored at -80°C until analysis of virus yields and the other fragment placed in RNALater stabilization reagent (Sigma-Aldrich) and also stored at -80°C until RNA extraction. The 1-lobulated left lung was processed for histopathological evaluation. Blood was collected at days 20, 42 and at sacrifice by submandibular bleeding, maintained at RT for 4 hours and centrifuged at 4°C for 20 min. at 3,600 rpm to obtain serum samples, which were then inactivated for 30 min. at 56°C and kept at -20°C until analyses of binding antibody titers and microneutralization test (MNT) assay were performed.

### 2.12 Processing of murine muscle for the analysis of immune cell recruitment

Muscle tissue was obtained during necropsy at days 1, 3 and 7 post-inoculations. For this, muscle from the inoculation site of each animal was dissected with a scalpel and placed in a 24-well plate containing complete Roswell Park Memorial Institute (RPMI) 1640 medium (Sigma-Aldrich; 100 U/mL penicillin/100 μg/mL streptomycin, 2 mM L-glutamine, 0.01 mM β-mercaptoethanol and 10 mM HEPES) with 10% heat-inactivated fetal calf serum (FCS; Sigma-Aldrich) and stored on ice until processing, following the protocol previously described (64). Briefly, muscle pieces were cut into smaller pieces (mechanical dissociation) and then incubated in 1 mL of digestion buffer [complete RPMI 1640 medium supplemented with 0.18 mg/mL Liberase TM (Roche, Basel, Switzerland), 40 μg/mL DNase I and 0.5 mg/mL collagenase VIII (both from Sigma-Aldrich)] for 40 min. in a shaking thermoblock at 37°C (enzymatic digestion). After, muscle samples were disaggregated and filtered, centrifuged, resuspended in incubation buffer (PBS 1X–EDTA 5 mM-3% FCS), cells counted and finally seeded on a 96-well plate according to the flow cytometry staining panels.

### 2.13 Processing of lung tissues for the analysis of SARS-CoV-2-specific immunogenicity

Lungs were harvested during necropsy and placed in a 24-well plate containing complete RPMI 1640 medium-10% FCS and stored on ice until processing. For this, lungs were transferred to an Eppendorf tube and divided into smaller pieces with a scalpel and tweezers (mechanical dissociation). Then, 1 mL of digestion buffer (complete RPMI 1640 medium supplemented with 0.18 mg/mL Liberase TM [Roche, Basel, Switzerland] and 40 μg/mL DNase I [Sigma-Aldrich]) was added and enzymatic digestion was performed by incubation for 40 min. in a shaking thermoblock (450 rpm at 37°C). After, digested lung samples were transferred to a Falcon tube containing 5 mL of stopping buffer (complete RPMI 1640 volume-3% FCS supplemented with 4 μg/mL DNase I) and final volume was filtered through 40-μm-pore cell strainers (BD Bioscience). The lung pieces were disaggregated on the filter with the help of the plunger of a 1 mL syringe while stopping buffer was passed. Lung samples were then centrifuged at 4°C for 10 min. at 1,640 rpm, supernatant harvested, and cells resuspended in 2 mL lysis buffer (0.1 M NH_4_Cl) and incubated for 2 min. at RT. After, lysis was stopped by adding incubation buffer (PBS 1X-EDTA 5 mM-3% FCS), cells filtered through 40-μm-pore cell strainers (BD Bioscience), centrifuged again at 4°C for 10 min. at 1,640 rpm, resuspended in 2 mL incubation buffer, cells counted and finally seeded on a 96-well plate to proceed with the staining for flow cytometry analysis.

### 2.14 Analysis of immune cell recruitment by flow cytometry

To evaluate the effect of CoV2-BMEP expression from DNA or MVA vectors on immune cell recruitment in muscle and DLNs, 1 × 10^6^ cells seeded on 96-well plates in incubation buffer were centrifuged for 3 min. at 2,000 rpm, washed once with incubation buffer and incubated with the LIVE/DEAD Fixable Red Dead Cell Stain Kit (Invitrogen) at 4°C for 30 min. in the dark. After washing once with incubation buffer and blocking Fc receptors with anti-CD16/CD32 antibody (BD Biosciences, San Jose, CA, USA) at 4°C for 5 min. in the dark, cells were incubated with MHC-II-biotin at 4°C for 20 min. in the dark. After washing once with incubation buffer, cells were incubated for 15 min. at 4°C in the dark with the following fluorochrome-conjugated surface antibodies for the recognition of different myeloid immune cell populations (BCs, NKs, TCs, moCs, NOs, EOs and DCs): Ly6G-phycoerythrin (PE), CD19-PE, CD3-PE, SinglecF-PE, Ly6C-peridinin chlorophyll protein (PerCP), avidin (Av)-PE-Cy7, CD64-allophycocyanin (APC), CD11b-Alexa Fluor 700, CD11c-APC-Cy7, CD45-PB and B220-BV510. All antibodies were from BD Biosciences. After washing once with incubation buffer, cells were transferred to cytometry tubes in incubation buffer and acquired in a Gallios flow cytometer (Beckman Coulter, Brea, CA, USA). Data analyses were performed using FlowJo software (version 10.4.2; Tree Star, Ashland, OR, USA). Cell-gated events ranged between 10^5^ and 5 × 10^5^. The gating strategies used for the identification of the different immune cell populations in muscle and DLNs are shown in **Supplementary Figure 1**.

### 2.15 Analysis of SARS-CoV-2-specific CD4 and CD8 T cell responses by flow cytometry

To determine the magnitude and phenotype of the SARS-CoV-2-specific T cell responses, 4 × 10^6^ splenocytes or 2 × 10^6^ lung-derived lymphocytes (both cell types were erythrocyte-depleted) seeded on 96-well plates were stimulated *ex vivo* for 6 h in complete RPMI 1640 medium with 10% FCS, 1 µL/mL Golgiplug (BD Biosciences), anti-CD107a-FITC (BD Biosciences), monensin 1X (Invitrogen) and 1 µg/mL of the different SARS-CoV-2 peptide pools representing the S, M and N antigens (JPT Peptide Technologies GmbH, Berlin, Germany). After stimulation, lymphocytes were stained for surface markers, fixed/permeabilized (Cytofix/Cytoperm kit; BD Biosciences) and intracellularly stained by incubation with the following fluorochrome-conjugated antibodies: IFN-γ-PeCy7, IL-2-APC and TNF-α-PE for functional analyses and CD3-PE-CF594, CD4-APC-Cy7 and CD8-V500 for phenotypic analyses (all from BD Biosciences). Dead cells were excluded from the analysis using the LIVE/DEAD Fixable Violet Dead Cell Stain kit (Invitrogen). Cells were acquired in a GALLIOS flow cytometer (Beckman Coulter), and data analyses were performed using FlowJo software (Version 10.4.2; Tree Star). Lymphocyte-gated events ranged between 10^5^ and 5 × 10^5^. Background responses obtained in unstimulated controls (RPMI) were subtracted from the responses detected in stimulated samples.

### 2.16 Measurement of SARS-CoV-2-specific binding and neutralizing antibodies

The presence of IgG, IgA and IgM binding antibodies against SARS-CoV-2 S and N proteins in serum was determined by Enzyme-Linked Immunosorbent Assay (ELISA) as previously described (65) using 2 μg/mL of recombinant SARS-CoV-2 S or N purified proteins (kindly provided by Dr. Casasnovas and Dr. Reyburn, respectively, both from CNB-CSIC, Madrid, Spain).

The capacity of the sera from immunized C57BL/6JOlaHsd mice or k18-hACE2 Tg mice to neutralize live SARS-CoV-2 virus (MAD6 isolate or the different VoCs Alpha, Beta, Delta and Omicron) was determined by a microneutralization test (MNT) assay performed in a BSL-3 laboratory at the CNB-CSIC as previously reported (66). Titers of NAbs were stablished as the reciprocal highest serum dilution that produced a 50% inhibition of cell death (neutralizing titer 50 [NT_50_]), following a protocol previously described (67).

### 2.17 Analysis of SARS-CoV-2 RNA and cytokines by reverse transcription-quantitative polymerase chain reaction

Lungs from infected SARS-CoV-2 k18-hACE2 Tg mice were harvested in RNALater (Sigma-Aldrich) and homogenized using a gentleMACS dissociator (Miltenyi Biotec, Bergisch Gladbach, Germany) in 2 mL of RLT buffer (Qiagen) with β-mercaptoethanol (Sigma-Aldrich). Samples were then processed for RNA isolation as previously described (66).

SARS-CoV-2 viral RNA was determined using previously validated probes and set of primers specific for the SARS-CoV-2 genomic RNA dependent RNA polymerase gene (*RdRp*) or the subgenomic E protein gene (*E*). The cellular 28S rRNA was used for normalization (68). For lung cytokine profile analysis, the above isolated RNA was used to determine the mRNA expression levels of *Cxcl10 (Ip-10), Il-6, Il-10, Tnf-alpha, Cxcl5* and *Ifit27* genes using TaqMan Gene Expression Assays (ThermoFisher Scientific, Waltham, MA, USA). Cellular 28S rRNA was used for normalization. Data were acquired in a 7500 real-time PCR system (Applied Biosystems, Waltham, MA, USA) and analysis was performed using the 7500 software v2.0.6. Relative RNA arbitrary units (A.U.) were determined relative to the negative group (group 3, non-infected mice) using the 2^−ΔΔCt^ method. Samples were evaluated in duplicate.

### 2.18 Analysis of SARS-CoV-2 virus yields by plaque assay

Lungs from k18-hACE2 Tg mice were collected, weighed, and homogenized with a gentleMACS dissociator (Miltenyi Biotec) in 2 mL of PBS buffer. Nasal turbinates were obtained after nasal washes with 0.1 mL of PBS. Virus yields in lung and nasal turbinates were determined as previously reported (66). SARS-CoV-2 titers were determined as PFUs per gram of lung tissue or PFUs per mL of nasal turbinate.

### 2.19 Lung histopathology

The complete 1-lobulated left lung was excised from each k18-hACE2 Tg mouse and immersion-fixed in 10% zinc formalin solution for 48 hours. After fixation, samples were processed and embedded in paraffin blocks that were sectioned in slides of 4 µm thickness on a microtome, mounted onto glass slides and stained with haematoxylin and eosin (H&E) (Histology Service, CNB-CSIC). Lung sections were analyzed using an Olympus BX43 microscope by a single veterinary pathologist (Veterinary Pathology Department, CISA-INIA) who was blind to the identity of mice as previously described (66). To determine the presence and severity of histopathological lesions, lung inflammation scores based on previous results on SARS-CoV-2 infection in mouse models were used (69). The histopathological parameters analyzed to obtain the corresponding lung inflammation score of each animal were graded according to a semi-quantitative scoring system as follows: (0) no lesion; (1) minimal lesion; (2) mild lesion; (3) moderate lesion; (4) severe lesion. The cumulative scores of histopathological lesions provided the total inflammation score per animal. The individual scores in each experimental group were used to calculate the group average.

### 2.20 Data analysis and statistics

For the statistical analysis of ICS data, an approach that adjusts the values for the non-stimulated controls (RPMI) and determines *p* values and confidence intervals was used (70). Only SARS-CoV-2 antigen responses significantly higher than the corresponding RPMI values were represented and background-subtracted. For the analysis of immune cell infiltration, ELISA, MNT, and virus yield data, we calculated the logarithm in base 10 of the values and used a 1-way ANOVA with the different groups as factor to determine the differences between them. If the ANOVA null hypothesis was rejected, we performed a *post-hoc* analysis with the Tukey’s honest significant difference criterion to determine which groups were significantly different from each other. For the comparison of the binding antibody and MNT titers within a group after prime and boost, we used a Student’s t-test on the logarithmically transformed data. For statistical analysis of lung histopathological lesion scores, the unpaired *t* test was used to establish differences among groups. Finally, for the statistical analysis of RT-qPCR data, unpaired non-parametric t tests with Welch´s correction was used to establish differences between groups. Statistical significances are indicated as follows: *, *p* < 0.05; **, *p* < 0.005; ***, *p* < 0.001.

## 3 Results

### 3.1 Characterization of the DNA vector expressing the multi-patch CoV2-BMEP protein

As described under Materials and Methods, we have used different computational and structural algorithms to characterize CoV2-BMEP, a novel multi-patch immunogen containing sequences from different structural proteins of SARS-CoV-2. The CoV2-BMEP (**Figure 1A**, **Supplementary Table 1**) linear sequence had a total length of 340 amino acids (~36 KDa) with a theoretical isoelectric point value (pI) of 8.87. The half-life was assessed to be 30 hours in mammalian reticulocytes *in vitro*, and >20 hours in yeast and >10 hours in *E. coli in vivo*. An instability index (II) of 26.84 was computed, classifying the protein as stable. The estimated aliphatic index was predicted to be 74.88, indicating thermostability. The predicted Grand average of hydropathicity (GRAVY) was −0.108, validating the hydrophilic nature of the construct. CoV2-BMEP was found to be non-allergenic by AllergenFP and antigenic by ANTIGENpro (0.855455).

3D structure predictions for CoV2-BMEP generally distinguished two separate N- and C-terminal domains, with the former being generally well-packed and composed mostly of alpha helices and the later being more unstructured and mainly composed by beta sheets. Interestingly, AlphaFold2 models also separate both domains but are generally less structured, with CABSfold model 6 looking somewhat similar yet showing better defined secondary structure and packing. In summary, the predicted CoV2-BMEP models suggest a relatively expanded, two-domain conformation. A representative model of the 3D structure of CoV2-BMEP construct is provided as an UCSF Chimera session (http://www.cgl.ucsf.edu/chimera; (71)) in **Figure 1B**.

The immunoinformatics analysis of CoV2-BMEP construct confirmed that the selected epitopes should be able to elicit antigenic responses in the designed sequence. The generated epitope/allele tables used to analyze the population coverage for eliciting T cell responses showed that 92% of the world population could be covered by MHC class I and II epitopes from CoV2-BMEP (**Supplementary Tables 2** and **3**).

Once CoV2-BMEP gene was synthesized and subcloned into the pcDNA vector, we analyzed the expression and subcellular localization of CoV2-BMEP from the resulting plasmid pcDNA-CoV2-BMEP (shortly DNA-CoV2-BMEP) by western-blotting and immunofluorescence analyses using a specific antibody against the HA tag located at the C-terminus of the CoV2-BMEP sequence. As shown in **Figure 1C**, CoV2-BMEP protein was detected in both pellet and supernatant samples of HEK293T transfected cells at the expected size (around 37 KDa) and at the different time-points evaluated, with the highest expression levels observed at 48h post-transfection. The different protein bands observed below 37 KDa likely correspond to the cleavage of the CoV2-BMEP construct by cellular proteases, due to the presence of the “fusion activation” proteolytic site S2′ (KPSKR815↓) in one of the S2 fragments included in the design. In transfected HeLa cells, CoV2-BMEP protein exhibited a diffuse and intense cytoplasmic distribution as visualized by confocal microscopy (**Figure 1D**).

### 3.2 *In vitro* characterization of MVA-CoV2-BMEP recombinant virus

#### 3.2.1 Purity

**Figure 2:**
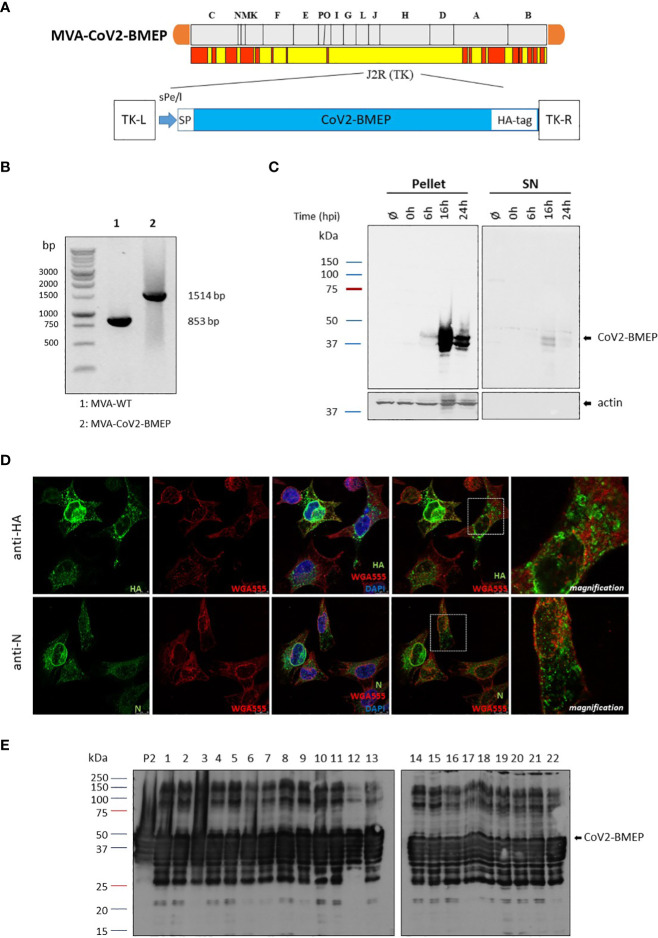
*In vitro* characterization of the MVA recombinant virus expressing the polyvalent multi-patch CoV2-BMEP protein. **(A)** Scheme of the thymidine kinase (TK) locus of MVA-CoV2-BMEP recombinant virus. **(B)** Confirmation of *CoV2-BMEP* gene insertion by PCR analysis. DNA was extracted from DF-1 cells infected with MVA-WT or MVA-CoV2-BMEP viruses at 5 PFU/cell. Primers TK-R and TK-L spanning TK flanking regions were used for the analysis of the TK locus by PCR. **(C)** Time-course expression of CoV2-BMEP protein by western-blotting analysis. HeLa cells were mock-infected or infected with MVA-CoV2-BMEP at 3 PFU/cell. At different times post-infection (0, 6, 16 and 24 h), cells were harvested and cellular pellets and supernatants (SN) were processed as described in Materials and Methods, fractionated by 12% SDS-PAGE and the expression of CoV2-BMEP protein was analyzed by western-blotting using a rabbit polyclonal anti-HA antibody. Actin was used as loading control. **(D)** Subcellular localization of CoV2-BMEP protein by confocal microscopy. HeLa cells were infected with MVA-CoV2-BMEP at 1 PFU/cell. At 16 h post-infection, cells were incubated with WGA-Alexa Fluor 594 (red staining), fixed and labeled with a rabbit polyclonal anti-HA antibody (upper panels) or a rabbit polyclonal anti-N antibody (lower panels). After incubation with the corresponding secondary antibody conjugated with Alexa Fluor 488 (green staining), cells were stained with DAPI (blue staining) to detect cell nuclei and visualized by confocal microscopy. **(E)** Analysis of the stability of the CoV2-BMEP protein expressed by MVA-CoV2-BMEP virus. Twenty-two individual plaques from MVA-CoV2-BMEP stability passage 7 were grown in DF-1 cells, lysed, proteins fractionated by 12% SDS-PAGE and analyzed by western-blotting using a rabbit polyclonal anti-HA antibody. The expression of CoV2-BMEP protein in cells infected with P2 stock or with individual plaques (1–22) is shown.

The *CoV2-BMEP* synthetic gene was inserted into the TK locus of MVA genome to generate the recombinant virus MVA-CoV2-BMEP (scheme in **Figure 2A**). The correct insertion of *CoV2-BMEP* gene into the viral genome and the purity of the MVA-CoV2-BMEP viral preparation were determined by PCR using primers annealing in the flanking regions of TK locus. As it is shown in **Figure 2B**, the *CoV2-BMEP* gene was successfully inserted into the TK locus of MVA genome since the expected 1514 bp product was detected and no parental MVA-WT contamination (853 bp product) was observed in MVA-CoV2-TMEP viral stock. These results were confirmed by DNA sequencing.

#### 3.2.2 Expression and Subcellular Localization of CoV2-BMEP Protein Expressed from MVA Vector

To confirm the expression of CoV2-BMEP construct from MVA vector over time we performed a Western-blotting analysis using pellet and supernatants (SN) of mock-infected or MVA-CoV2-BMEP-infected HeLa cells. As shown in **Figure 2C**, increasing amounts of CoV2-BMEP protein at the expected 37 KDa size was detected over time in the pellet of MVA-CoV2-BMEP-infected cells, while in the supernatant fractions it was detected at late time-points (16 h.p.i.). Actin was used as loading control. By confocal microscopy using anti-HA or anti-N specific antibodies we detected that CoV2-BMEP protein formed aggregates in the cytoplasm of infected cells (**Figure 2D**).

#### 3.2.3 Genetic Stability

A requirement for a successful manufacture of MVA-based recombinant vectors is to demonstrate the genetic stability of the heterologous gene within the viral genome. Therefore, to ensure that *CoV2-BMEP* gene inserted into the parental TK locus of MVA genome was stably integrated and can be maintained in the viral genome without sequence modifications, 7 successive infection passages in DF-1 cells infected with MVA-CoV2-BMEP recombinant virus at low multiplicity of infection were performed. Then, passage 7 was used to infect DF-1 cells and 22 individual plaques were picked up, grown in DF-1 cells and western-blotting analysis was performed with infected cell extracts to detect the expression of CoV2-BMEP protein in each individual plaque. As shown in **Figure 2E**, 22 out of 22 plaques (100% stability) isolated from passage 7 correctly expressed the CoV2-BMEP protein, indicating that the insertion of the heterologous gene was highly stable. Higher molecular size proteins were also observed that could represent oligomeric forms.

#### 3.2.4 CoV2-BMEP recognition by CD8 T lymphocytes from SARS-CoV-2 convalescents

**Figure 3:**
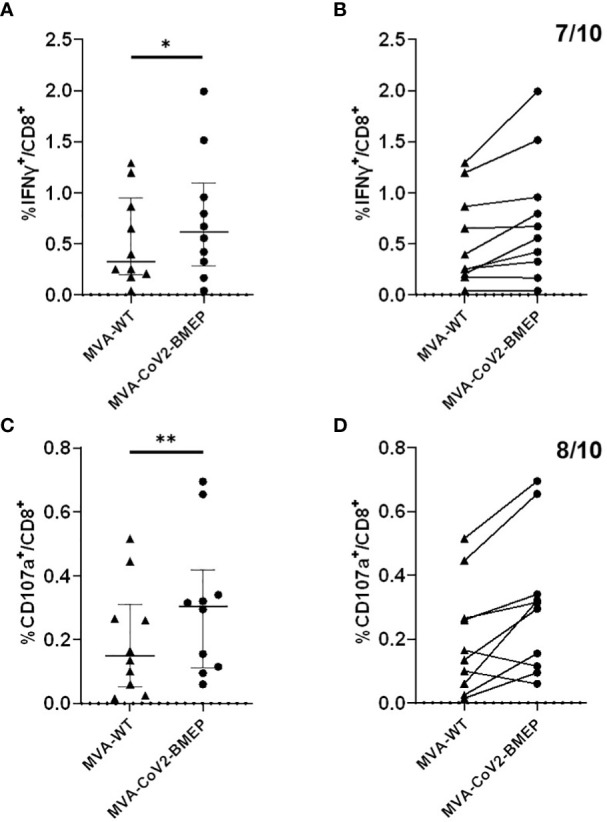
Human CD8^+^ T lymphocyte response against CoV2-BMEP. Immune response to autologous cells infected with MVA-WT or MVA-CoV2-BMEP of 10 healthy volunteers who had tested positive for SARS-CoV-2. The percentage of CD8^+^ T lymphocytes producing IFN-γ or expressing CD107a as a measure of activation is represented. **(A, B)** show the IFN-γ response against both viruses (after subtracting the background of mock uninfected cells). **(A)** represents the individual IFN-γ response and the median and interquartile range of each group, while **(B)** links with a line the individual response of each volunteer against both viruses; seven of the ten volunteers respond to MVA-CoV2-BMEP. **(C, D)**, same as **(A, B)** but showing the CD107a response of CD8^+^ T lymphocytes to the two viruses. Eight of the ten volunteers respond to MVA-CoV2-BMEP. Statistical analysis: Paired t test, *,p=0.017; **,p=0.0094.

Since the patches from the SARS-CoV-2 structural proteins included in the synthetic CoV2-BMEP construction contain functional CTL epitopes, we decided to evaluate *in vitro* the recognition of CoV2-BMEP by CD8 T cells from SARS-CoV-2 convalescents. For this, PBMCs from 10 SARS-CoV-2-infected and fully recovered patients were stimulated with autologous cells infected with MVA-WT or MVA-CoV2-BMEP viruses as described under Materials and Methods. The CoV2-BMEP-specific CD8 T cells secreting IFN-γ or CD107a were determined by FACS. As shown in **Figure 3**, 70%-80% of the convalescents were able to specifically recognize the synthetic protein when expressed from MVA-CoV2-BMEP. Seven out of ten volunteers exhibited effector IFN-γ response of CD8^+^ T lymphocytes to MVA-CoV2-BMEP (**Figures 3A, B**), while eight out of ten exhibited cytotoxic CD8 T cell responses using the indirect degranulation marker CD107a (**Figures 3C, D**). Overall these data suggest that the synthetic CoV2-BMEP protein is processed in MVA-CoV2-BMEP-infected cells allowing its recognition by CD8 T cells from naturally infected SARS-CoV-2 survivors.

### 3.3 Effect of CoV2-BMEP expression on immune cell recruitment in muscle and DLNs

**Figure 4:**
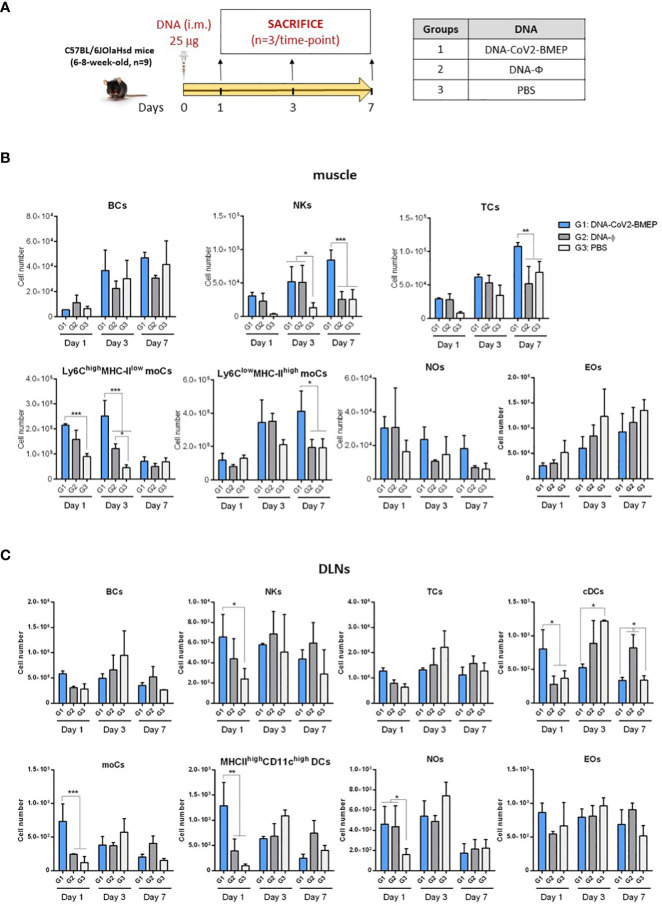
Innate immune response elicited in muscle and DLNs by the DNA vector expressing the polyvalent multi-patch CoV2-BMEP protein. **(A)** Immunization schedule. Three groups of animals were inoculated with 25 μg of DNA-CoV2-BMEP or DNA-φ or PBS by the intramuscular (i.m.) route. At days 1, 3 and 7 post-inoculation, total muscle from the site of inoculation and DLNs were excised and processed as described in Materials and Methods. **(B, C)** Different immune cell populations present in muscle **(B)** and DLN **(C)** cell suspensions determined by flow cytometry. BCs, B cells; NKs, Natural killer cells; TCs, T cells; moCs, Monocyte-derived cells; NOs, Neutrophils; EOs, Eosinophils; DCs, Dendritic cells; cDCs, Conventional dendritic cells. Data are shown as mean and SD. *, *p* < 0.05; **, *p* < 0.005; ***, *p* < 0.001.

**Figure 5:**
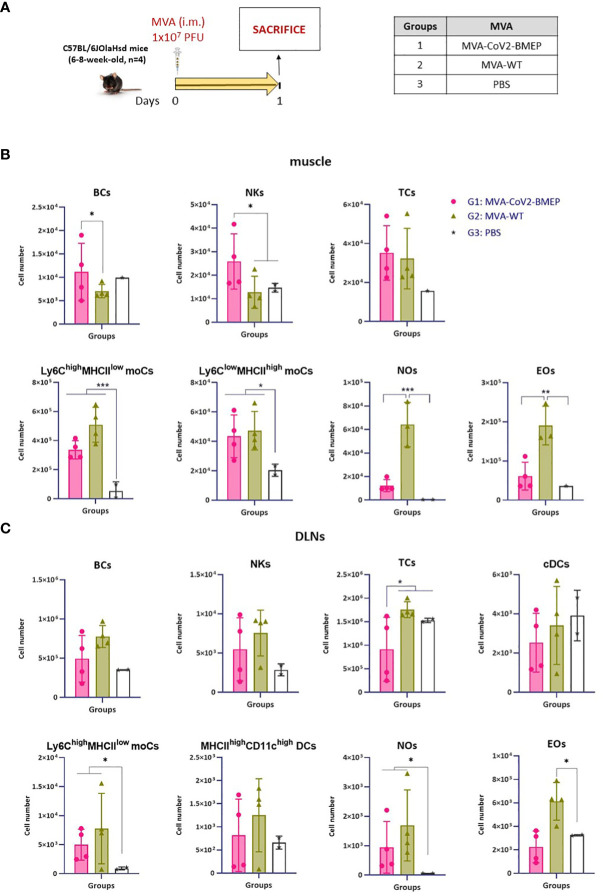
Innate immune response elicited in muscle and DLNs by the MVA vector expressing the polyvalent multi-patch CoV2-BMEP protein. **(A)** Immunization schedule. Three groups of animals were immunized with 1 x 10^7^ PFU of MVA-WT or MVA-CoV2-BMEP or PBS by the i.m. route. At day 1 post-inoculation, total muscle from the site of inoculation and DLNs were excised and processed as described in Materials and Methods. **(B, C)** Different immune cell populations present in muscle **(B)** and DLN **(C)** cell suspensions determined by flow cytometry. Data are shown as colored forms for each animal with mean and SD. *, *p* < 0.05; **, *p* < 0.005; ***, *p* < 0.001.

We next evaluated how the expression of CoV2-BMEP protein from DNA or MVA vectors impacts on the recruitment of immune cells in the muscle and DLNs of vaccinated mice. After gating on CD45^+^ cells and exclusion of cells expressing CD3 (T cells -TCs-), CD19 (B cells -BCs-), SinglecF (eosinophils -EOs-) or Ly6G (neutrophils -NOs-), a sequential gating strategy based on the differential expression of CD64, Ly6C and MHC-II allowed the characterization of Ly6C^high^ MHC-II^low^ and Ly6C^low^ MHC-II^high^ monocyte-derived cells (moCs) (**Supplementary Figure 1**). The immunization schedules and the different immunization groups are shown in **Figures 4A**, **5A**.

In muscle cells from animals injected with DNA vectors we observed a continous increase in the number of BCs and EOs from day 1 to 7 that could be associated with tissue injury caused by the inoculation process, since no differences are observed between groups (**Figure 4B**). Regarding the number of NOs, an increase in cell number from DNA-primed groups at day 1 was detected compared to PBS-treated animals. This increase, in contrast to BCs and EOs, progressively decreases at days 3 and 7. Interestingly, the local expression of CoV2-BMEP protein induced a significant increase in the number of Ly6C^high^ MHCII^low^ moCs, differentiated from monocytes recruited at early time-points after immunization that peaked at day 3. This subpopulation progressively downregulated the expression of Ly6C and upregulated MHC-II, generating the Ly6C^low^ MHC-II^high^ moCs subpopulation, that was significantly higher at day 7 in mice immunized with DNA-CoV2-BMEP vector. In DLNs the inoculation of DNA-CoV2-BMEP induced an early inflammatory response at day 1 characterized by the recruitment of natural killer (NK) cells and NOs, and a significant increase in the number of moCs differentiated from recruited monocytes and resident conventional dendritic cells (cDCs), probably mediated by the recruitment of cDC-precursor cells (preDCs) (**Figure 4C**). DNA-CoV2-BMEP immunization also induced an incresase in the number of MHC-II^high^ CD11c^high^ DCs that phenotipycally resemble tissue-derived DCs that most probably have migrated from the muscle to the lymph node.

When MVA vector was used for animal inoculation we observed that CoV2-BMEP protein expression in muscle cells was able to recruit higher numbers of BCs and NKs (**Figure 5B**), whereas in DLNs similar numbers of innate immune cell populations were detected in MVA-CoV2-BMEP-immunized mice and in MVA-WT control group (**Figure 5C**). This could be due to the pro-inflammatory profile induced *per se* by the MVA vector. It should be noted the high levels of NOs and EOs observed in both muscle and DLNs in animals immunized with MVA-WT vector.

In summary, the multi-patch CoV2-BMEP protein delivered *in vivo* by DNA and MVA vectors triggered an increase in the recruitment of a variety of immune cells with critical roles in innate and adaptive immune responses.

### 3.4 SARS-CoV-2-specific adaptive immune responses induced in mice by homologous or heterologous prime/boost immunization protocols using DNA or MVA vectors expressing CoV2-BMEP protein

**Figure 6:**
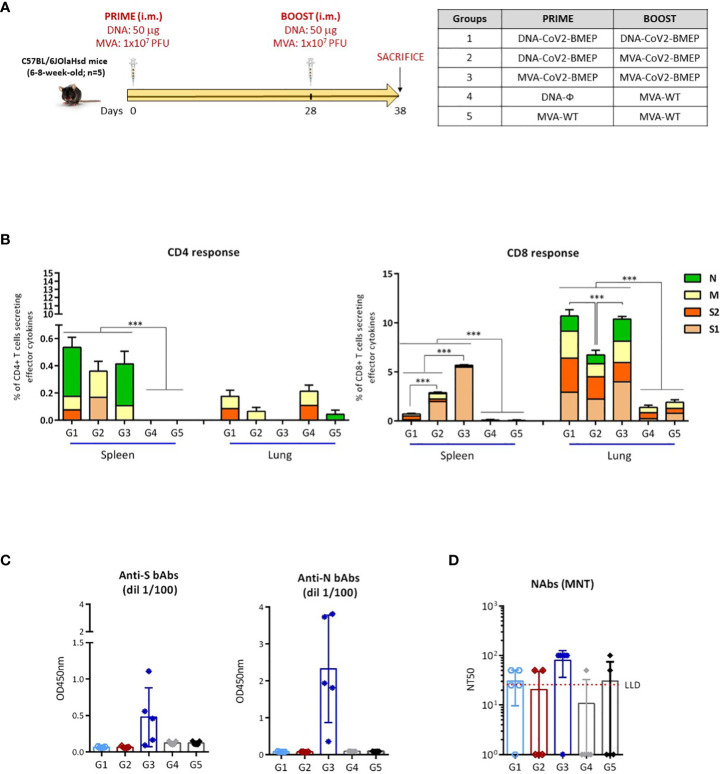
Cellular and humoral adaptive immune responses elicited by DNA-CoV2-BMEP and MVA-CoV2-BMEP in C57BL6 mice when administered in homologous or heterologous regimens. **(A)** Immunization schedule. **(B)** Magnitude of the total SARS-CoV-2-specific CD4 (left) or CD8 (right) T cells at 10 days post-boost by ICS assay after the stimulation of lymphocytes derived from spleen or lung with SARS-CoV-2 peptide pools. The total value of each group represents the sum of the percentages of SARS-CoV-2-specific CD4 or CD8 T cells secreting IFN-γ and/or IL-2 and/or TNF-α against SARS-CoV-2 peptide pools. Data are background-subtracted. 95% CI is represented. ***, *p* < 0.001. **(C)** Level of binding antibodies to SARS-CoV-2 S (left) or N (right) proteins from Wuhan reference strain elicited in serum from immunized individual mice at 10 days post-boost measured as OD_450_ at a serum dilution of 1:100 by ELISA. Data are shown as colored forms for each animal with mean and SD. **(D)** Level of neutralizing antibodies to SARS-CoV-2 (Wuhan strain) elicited in serum from immunized individual mice at 10 days post-boost measured as NT50 by microneutralization assay. Data are shown as colored forms for each animal with mean and SD. The red dashed line represents the lower limit of detection (LLD) of the assay.

Next, we characterized in mice the SARS-CoV-2-specific immunogenicity elicited by DNA or MVA vectors expressing the CoV2-BMEP protein when administered in homologous or heterologous combinations. The immunization schedule and the different immunization groups are shown in **Figure 6A**.

#### 3.4.1 SARS-CoV-2-Specific T Cell immune responses

At 10 days post-boost, lymphocytes derived from spleen or lung of immunized mice were stimulated *ex vivo* for 6 h with SARS-CoV-2 peptide pools and incubated with specific antibodies to identify T cell lineage (CD3, CD4 and CD8) and effector cytokines (IFN-γ, IL-2 and TNF-α) and degranulation (CD107a) to define responding cells. SARS-CoV-2-specific CD4 and CD8 T cells were determined by the percentage of T cells with CD4 or CD8 phenotype that produced IFN-γ and/or IL-2 and/or TNF-α.

As shown in **Figure 6B**, SARS-CoV-2-specific T cell response was polarized towards the CD8 compartment in all immunization groups. For CD4 T cells, SARS-CoV-2-specific responses in spleen were higher in mice immunized with the different homologous or heterologous combinations of vectors expressing CoV2-BMEP protein compared to control groups, although no statistical differences were obtained between the different immunization regimens. CD4^+^ T cell responses in spleen were mainly directed against N and M peptide pools. No SARS-CoV-2-specific CD4 T cell responses above control groups were observed in lung. Probably the frequency of CD4-specific T cells was low and the sensitivity of our assay did not allow us to detect it. For CD8 T cells, responses obtained in lung were considerably higher than those observed in spleen. The highest SARS-CoV-2-specific CD8 T cell response in spleen was detected in mice immunized with the homologous combination MVA-CoV2-BMEP/MVA-CoV2-BMEP and this response was directed exclusively against S1 peptide pool. In lung, the highest SARS-CoV-2-specific CD8 T cell responses were observed in mice immunized with the homologous combinations of vectors (DNA-CoV2-BMEP/DNA-CoV2-BMEP or MVA-CoV2-BMEP/MVA-CoV2-BMEP) and this response was evenly distributed between S1, S2, M and N peptide pools.

#### 3.4.2 SARS-CoV-2-Specific humoral immune responses

Next, we characterized the humoral responses (binding antibodies against SARS-CoV-2 S and N proteins and neutralizing antibodies against SARS-CoV-2 virus) induced by CoV2-BMEP protein expressed from DNA or MVA vectors at 10 days post-boost. The reactivity of serum from individual mice against the purified SARS-CoV-2 S and N proteins was quantified by ELISA. As shown in **Figure 6C**, the group of mice immunized with the homologous combination MVA-CoV2-BMEP/MVA-CoV2-BMEP was the only regimen able to induce significant binding antibodies levels against S and N proteins. The same result was observed when we analyzed the neutralization capacity against SARS-CoV-2 virus of the sera from immunized mice. As shown in **Figure 6D**, the combination MVA-CoV2-BMEP/MVA-CoV2-BMEP was the best immunization regimen for the induction of SARS-CoV-2-specific neutralizing antibodies.

### 3.5 Efficacy study in humanized k18-hACE2 Tg mice

**Figure 7:**
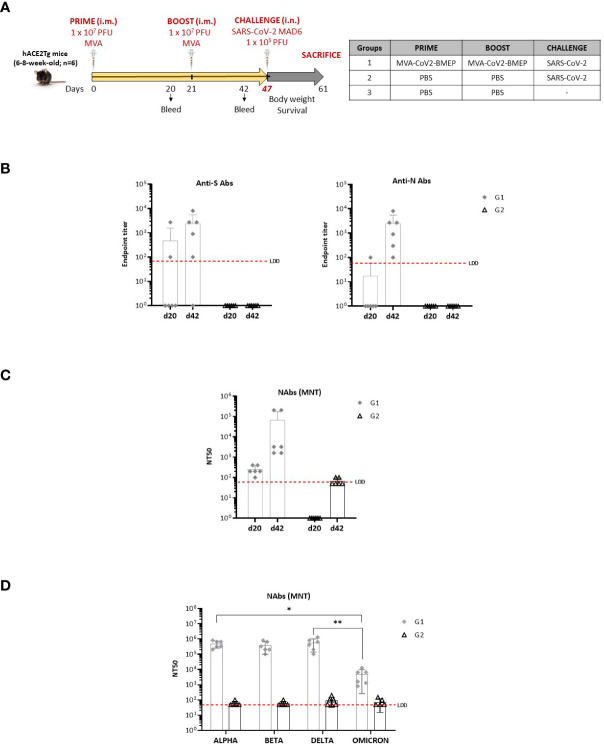
Efficacy study in humanized k18-hACE2 Tg mice. **(A)** Immunization schedule. **(B)** Level of binding antibodies to SARS-CoV-2 S (left) or N (right) proteins from Wuhan reference strain elicited in serum from immunized individual mice at days 20 (post-prime) and 42 (21 days post-boost) before challenge. The endpoint titer represents the last serum dilution that gave 3 times the mean OD_450_ value of the control group. Data are shown as forms for each animal with mean and SD. The red dashed line represents the lower limit of detection (LLD) of the assay. **(C, D)** Titers of SARS-CoV-2 neutralizing antibodies (NT50) in serum from immunized individual mice against SARS-CoV-2 MAD6 isolate **(C)**, or serum from two immunized mice with the highest NT50 against Alpha, Beta, Delta and Omicron variants **(D)** determined in triplicates using a live virus MNT assay at days 20 (post-prime) and 42 (21 days post-boost) for MAD6 isolate or d42 for SARS-CoV-2 variants before challenge. Data are shown as forms for each animal with mean and SD. The red dashed line represents the LLD of the assay. *, *p* < 0.05; **, *p* < 0.005.

Since the group of MVA-CoV2-BMEP administered in homologous combination was the best one that elicited both binding and neutralizing antibodies against SARS-CoV-2, we selected this regimen for efficacy studies. The immunization schedule and the different immunization groups are shown in **Figure 7A**.

#### 3.5.1 Analysis of the humoral immune responses at Pre-Challenge

We characterized the humoral responses (binding antibodies against SARS-CoV-2 S and N proteins and neutralizing antibodies against SARS-CoV-2 virus) induced by MVA-CoV2-BMEP at 20 days post-prime (d20) and 21 days post-boost (d42). The reactivity of serum from individual mice against the purified SARS-CoV-2 S and N proteins was quantified by ELISA at both time-points. As shown in **Figure 7B**, the group of mice immunized with one dose of MVA-CoV2-BMEP (d20) induced levels of binding antibodies against SARS-CoV-2 S and N proteins above control group, and these levels were enhanced after a second dose of MVA-CoV2-BMEP (d42), reaching endpoint titers in the range of 10^2^ – 10^4^. An increase in the systemic levels of anti-S IgA and IgM induced by MVA-CoV2-BMEP vector at d20 and d42 was also detected (**Supplementary Figure 2**). The same result was observed when we analyzed the neutralization capacity against SARS-CoV-2 virus of serum from immunized mice. As shown in **Figure 7C**, one dose of MVA-CoV2-BMEP (d20) induced SARS-CoV-2-specific neutralizing antibodies and these levels were boosted after a second dose of MVA-CoV2-BMEP (d42), reaching NT50 titers in the range of 10^3^ - 10^5^. The six vaccinated animals developed neutralizing antibodies but these levels were markedly enhanced in two animals of the group. We selected these two serum samples for further characterization of the extent of neutralizing capacity. In fact, serum from these animals cross-neutralized different SARS-CoV-2 VoCs (Alpha, Beta, Delta and Omicron) before coronavirus challenge (d42) (**Figure 7D**), with values of NT50 in the range of 10^5^ – 10^6^ for Alpha, Beta and Delta variants and of 10^3^ – 10^4^ for Omicron.

#### 3.5.2 Partial protection from morbidity and mortality of two doses of MVA-CoV2-BMEP in humanized k18-hACE2 Tg mice challenged with SARS-CoV-2 virus

**Figure 8:**
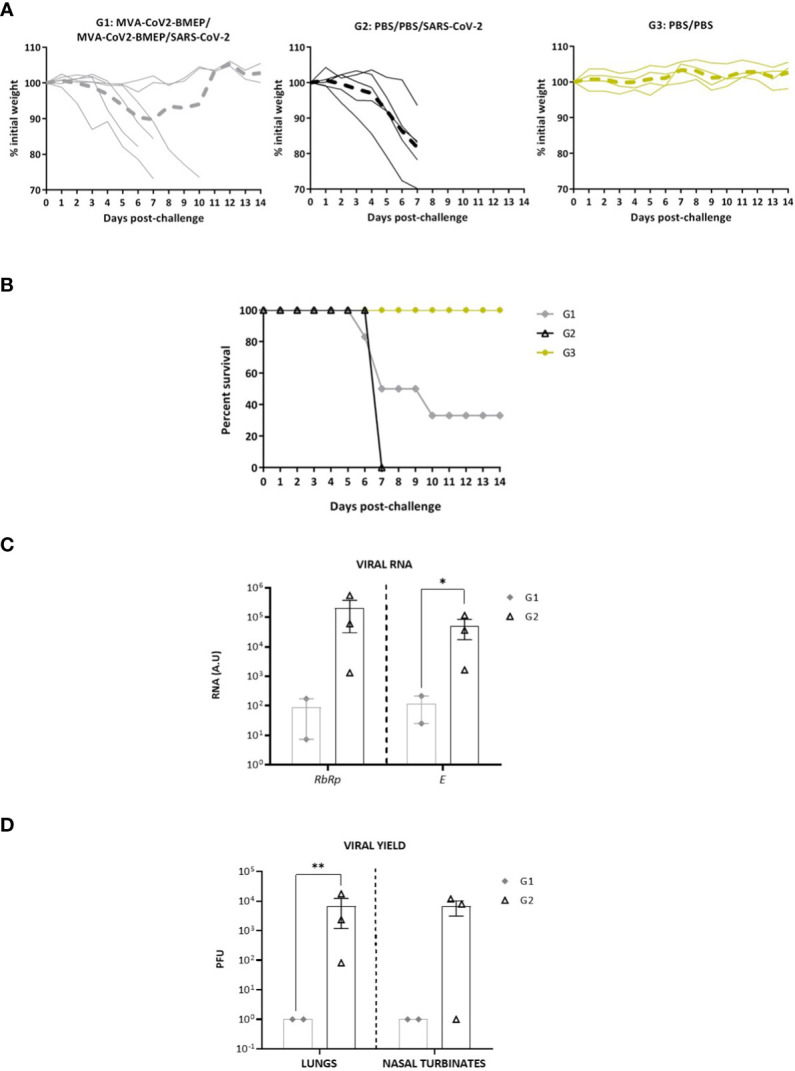
Two doses of MVA-CoV2-BMEP partially protect from morbidity and mortality in humanized k18-hACE2 Tg mice infected with SARS-CoV-2. The challenged mice were monitored for body weight variations **(A)** and mortality **(B)** for 14 days. **(C)** Virus replication in lung samples. Genomic (*RdRp*) and subgenomic (*E*) SARS-CoV-2 RNAs were detected by RT-qPCR after virus challenge in surviving mice. RNA levels [in arbitrary units (A.U.)] for each animal are represented as forms with mean and SD from duplicates; relative values are referred to non-challenged PBS-inoculated mice (group 3). *, *p* < 0.05. **(D)** SARS-CoV-2 infectious virus in lung samples and nasal turbinates. Data are shown as forms for each animal with mean (PFUs/g of lung tissue or PFUs/mL of nasal turbinate) and SD from duplicates. **, *p* < 0.005.

Next, we characterized the efficacy of vaccination after challenge with live SARS-CoV-2 virus. As it is observed in panels A and B of **Figure 8**, all PBS-treated non-challenged mice (group 3) maintained body weight while PBS-treated challenged mice (group 2) lost body weight and had to be sacrificed at 7 days post-challenge (p.c.). In the group of mice vaccinated with two doses of MVA-CoV2-BMEP and challenged with SARS-CoV-2 virus (group 1), 2 out of 6 (33.33%) mice did not lose body weight and survived until the end of the study (day 14 p.c.). The two surviving animals were those with the highest neutralizing antibodies titers at 20 days post-prime (d20) and 21 days post-boost (d42) at pre-challenge (**Figure 7C**). The other four animals of the MVA-CoV2-BMEP group lost body weight and had to be sacrificed at 6 (1 animal), 7 (2 animals) and 10 (1 animal) days p.c.

#### 3.5.3 Restricted SARS-CoV-2 virus replication by vaccination

Analysis of SARS-CoV-2 virus replication at days 7 (group 2) or 14 (group 1) post-challenge in lung samples showed that, in surviving animals from MVA-CoV2-BMEP group, SARS-CoV-2 virus replication (genomic *RdRp* and subgenomic *E* mRNAs) was significantly reduced compared to unvaccinated challenged control group (**Figure 8C**). This observation correlated with the analysis of viral yields in lung homogenates and nasal turbinates (**Figure 8D**), where mice immunized with MVA-CoV2-BMEP showed about 4-log reduction of infectious virus compared to challenged control mice.

#### 3.5.4 Lung histopathological and cytokine profile analyses of mice vaccinated with MVA-CoV2-BMEP and challenged with SARS-CoV-2

**Figure 9:**
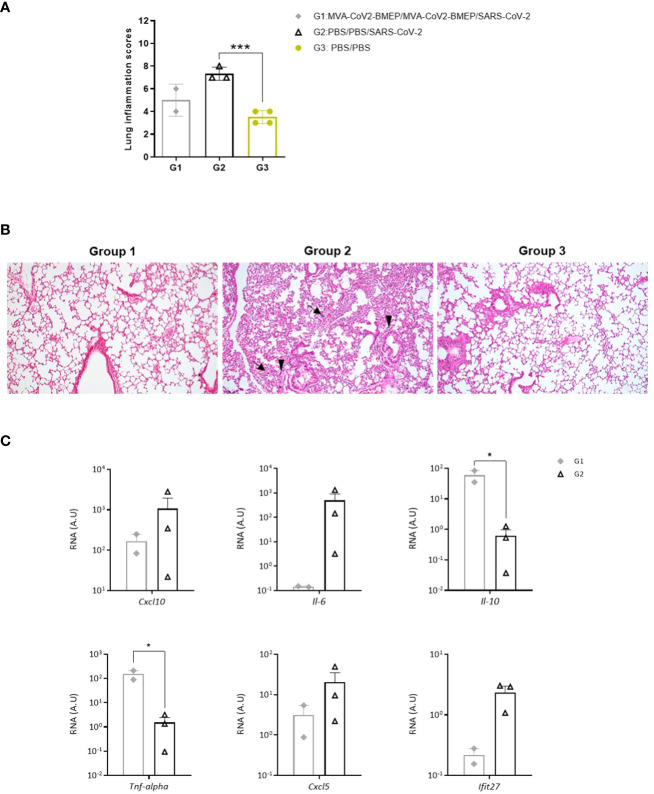
Lung histopathological and cytokine profile analyses of mice vaccinated with MVA-CoV2-BMEP and challenged with SARS-CoV-2. **(A)** Lung inflammation scores obtained in lung samples from individual mice immunized with MVA-CoV2-BMEP, challenged with SARS-CoV-2 and euthanized at 14 days post-challenge (group 1; n=2), PBS-treated mice challenged with SARS-CoV-2 and euthanized at 7 days post-challenge (group 2; n=3) and PBS-treated non-challenged mice euthanized at 14 days post-challenge (group 3; n=4). Data are represented as colored forms for each animal with mean and SD of cumulative histopathological lesions for each experimental group. ***, *p* < 0.001. **(B)** Representative lung histopathological sections from k18-hACE2 Tg mice included in experimental groups 1, 2 and 3. Mice in groups 1 and 3 did not display remarkable inflammatory lesions while mice in group 2 showed inflammatory lesions such as diffuse mild to moderate thickening of the alveolar septa, diffuse mild alveolar mononuclear cell infiltrates (arrows) or the presence of ocassional multifocal perivascular and peribronchiolar mononuclear infiltrates (arrowheads). H&E staining; Magnification: 10x. **(C)** Levels of RNA from pro-inflammatory cytokines and chemokines genes detected by RT-qPCR. Targeting genes (*Cxcl10, Il-6, Il-10, Tnf-alpha, Cxcl5* and *Ifit27*) were normalizated to cellular 28S rRNA in lungs obtained at 14 days post-challenge in surviving mice. RNA levels (in A.U.) for each animal are represented as forms with mean and SD from duplicates; relative values are referred to non-challenged PBS-inoculated mice (group 3). *, *p* < 0.05.

Next, we analyzed the histopathological changes observed in lungs from immunized mice. As shown in **Figure 9A**, the lung lesion scores observed in animals from groups 1 (MVA-CoV2-BMEP/MVA-CoV2-BMEP/SARS-CoV-2) and 3 (non-challenged PBS-treated mice) were similar and lower than those described in mice from group 2 (PBS/PBS/SARS-CoV-2). One mouse in group 1 also displayed focal areas with well consolidated bronchus associated lymphoid tissue (BALT). Among the pulmonary lesions characteristic of SARS-CoV-2 infection observed in mice from group 2, highlighted the presence of multifocal mild alveolar haemorrhages, diffuse mild alveolar mononuclear infiltrates, occasional bronchi and bronchioles with detached epithelium or inflammatory mononuclear cells in the lumen (bronchitis or bronchiolitis), ocassional mild to moderate perivascular oedema, diffuse mild to moderate thickening of alveolar septa and multifocal mild perivascular and peribronchial/peribronchiolar mononuclear infiltrates (**Figure 9B**).

Finally, since up-regulation of several pro-inflammatory cytokines has been correlated with COVID-19 disease progression and severity (72–74), we analyzed the effect of MVA-CoV2-BMEP immunization on the cytokine expression profile induced in infected mice. Thus, at 7 (group 2) or 14 (group 1) days post-challenge, mRNA levels of key cytokines were evaluated by RT-qPCR in lung homogenates from immunized mice. The results showed that two doses of MVA-CoV2-BMEP down-regulated *Cxcl10 (Ip-10), Il-6, Cxcl5* and *Ifit27* mRNA levels and significantly up-regulated mRNAs levels of *Il-10* and *Tnf-α* compared to control challenged mice (**Figure 9C**).

## 4 Discussion

Vaccines against the coronavirus SARS-CoV-2 are mostly based on a vector delivery system of the S (spike) protein of the virus, a trimeric glycoprotein expressed on the surface of the virion that is the only viral protein known to target for NAbs. It comprises an apical S1 subunit containing an N-terminal domain (NTD) and the receptor-binding domain (RBD), which is responsible for the binding to the ACE2 receptor; and a membrane-proximal S2 subunit, responsible for the fusion of cellular and viral membranes. In Europe, the approved vaccines against SARS-CoV-2 consist of modified mRNA-S formulated in lipid nanoparticles, adenovirus vectors expressing the S antigen or adjuvanted S protein. The clinical studies have provided solid evidence that the administration of these vaccines in humans efficiently protects against severe disease, hospitalizations and death across age groups and in diverse populations (75–81). However, this protection was maintained for a limited time, generally about 6 months, and was affected by the emergence of SARS-CoV-2 VoCs, being the neutralization response more prone to decay compared with the cellular immunity (13, 82). For this reason, the regulatory authorities (FDA and EMA) and WHO have recommended the administration of booster doses in order to prolong the duration of immune responses and to increase the protection capacity against highly mutated VoCs. As such, the susceptible population in developed countries is currently receiving a fourth booster dose of the SARS-CoV-2 vaccine. The main question is why current vaccines confer limited time of protection against infection. There are several reasons: i) these vaccines induced NAbs, but they decay with time and become less effective; ii) antibodies produced by the vaccines poorly neutralized some of the VoCs, like Beta and Omicron; iii) the vaccines do not efficiently induce mucosal immune responses, particularly IgA, since the administration by i.m. route triggers mainly IgGs; iv) protective B and T cell responses are only directed against the S viral antigen and not against other structural and non-structural proteins that could contribute to increase antibody-based protection and long-term durability of vaccine-induced responses and; v) the current vaccines do not fully protect against virus transmission and reinfections.

In this investigation, we have attempted to broaden the capacity of vaccines based on the single S protein through the design of a polyvalent multi-patch immunogen containing conserved regions from the SARS-CoV-2 S, M and N structural proteins enriched in B and T cell epitopes that have been functionally associated with protection in SARS-CoV and SARS-CoV-2 convalescents. Polyvalent vaccines are less vulnerable to antigenic-drift and virus evasion after immune pressure than monovalent vaccines currently approved. For this reason, we have produced a codon-optimized synthetic sequence (1514 bp; referred as CoV2-BMEP) that was incorporated in either DNA or MVA vectors. The functional CTL epitopes included in CoV2-BMEP design were recognized *in vitro* by specific CD8 T cells from SARS-CoV-2 convalescents. The magnitude of the human response, although moderate, was within the same range that we observe in responses to full length N and M proteins in infected patients (unpublished data). It is noteworthy that, as in our set of patients, it is a common observation that CD8 T cell responses are not detected in all SARS-CoV-2-infected individuals (for example, in 70% of patients in Grifoni et al. (83)). The presence of specific response evidences that CoV2-BMEP is processed in human cells, generating epitopes that are present in the natural response against the virus.

In cultured cells, both platforms efficiently expressed CoV2-BMEP as a main protein of about 37 kDa, together with other processed products due to the presence of the “fusion activation” proteolytic site S2′ in one of the S2 region included in the design. Different bNAbs recognizing the FP epitope have been isolated from SARS-CoV-2 immune donors (84–86). These bNAbs bound to the shared _815_RSFIEDLLF_823_ motif located within the SARS-CoV-2 FP region at the C-terminal of the S2’ cleavage site. Importantly, the arginine at the S2’ cleavage site (R_815_) is a critical residue involved in the recognition by these anti-FP antibodies, suggesting that binding to the S2’ cleavage site may be a distinguishing property of broadly neutralizing mAbs against this site. For this reason, we decided to maintain the intact epitope as part of the CoV2-BMEP S_808-835_ region. The FP region is well-recognized by sera from convalescent donors but not by sera from people vaccinated with mRNA or Ad-based vaccines expressing the S protein stabilized in the pre-fusion state by using 2P (K986P, V987P) mutations. This is consistent with a greater exposure of the S2 subunit to B cells during natural infection due to S1 uncoupling and with the fact that the 2P mutations abolish the receptor-induced exposure of the fusion peptide (85). Considering that the FP region is highly conserved among all genera of the Orthocoronavirinae subfamily, the insertion of the intact region with the S2’ site in our design could benefit the induction of cross-specific bNAbs that could both prevent S proteolytic activation or fusogenic rearrangements, thereby inhibiting membrane fusion and viral entry and TMPRSS2 cleavage of the S2’ site (via steric hindrance) and in turn activation of membrane fusion.

Both DNA-CoV2-BMEP and MVA-CoV2-BMEP vectors were able to recruit immune cells to the site of inoculation and to the draining lymph nodes when administered *in vivo* by intramuscular route, some of them clearly implicated in antigen presentation and activation of adaptive immune responses.

In mice immunized with DNA-CoV2-BMEP and MVA-CoV2-BMEP vectors there was an induction of SARS-CoV-2-specific B and T cell responses, and these responses were higher and broader with the homologous MVA/MVA prime/boost immunization protocol than with the DNA/DNA regimen. In particular, for the homologous MVA/MVA combination the specific CD8^+^ T cell responses in the lung were of high magnitude and broadly distributed against the SARS-CoV-2 S1, S2, M and N proteins, while in the spleen the main CD8 T cell activation was mediated by the S1 domain. The induction of specific CD8^+^ T cells in lung after systemic homologous administration of DNA/DNA or MVA/MVA vectors may be critical for protection, since the lungs are the primary site for the development of COVID-19. We could not detect SARS-CoV-2-specific CD4 T cell responses in lung above control groups, although in spleen we were able to detect both CD4 and CD8-specific T cells. Other studies using MVA vectors expressing the full-length S protein were unable to detect CD4^+^ T cells in the lung of inoculated animals or reported low levels of CD4 T cells specific against the S1 domain of the SARS-CoV-2 spike protein, but not against the S2 domain that contains the majority of the conserved S region included in our immunogen (66, 87, 88).

Considering the higher levels of binding antibodies against S and N viral proteins and the broad activation of specific CD4 and CD8 T cell responses induced by the homologous MVA/MVA combination compared with the DNA/DNA regimen, we selected MVA-CoV2-BMEP vector for further efficacy studies. In susceptible transgenic mice for human ACE2 receptor, two doses of MVA-CoV2-BMEP induced binding antibodies against S and N proteins and NAbs that increased in magnitude after the booster dose. In terms of morbidity and mortality, while all animals in the control group lost weight and died at day 7 p.c., two animals out of six vaccinated mice did not lose weight and survived until the end of the assay (day 14 p.c.). This protection correlated with the induction of high levels of NAbs, with strong reduction (3-logs) of genomic and subgenomic SARS-CoV-2 RNA in the lung tissue, with severe reduction (4-logs) of virus yields in lung and nasal turbinates, with lung lesion scores lower than those described in mice treated with PBS and challenged with SARS-CoV-2 and also with reduction in the RNA levels of some pro-inflammatory cytokines and chemokines. Moreover, serum from protected animals also cross-neutralized different SARS-CoV-2 VoCs, including Omicron, that continued to be the dominant virus globally. NAbs levels detected in protected animals by live virus microneutralization assay (10^4^-10^5^) were comparable or even higher than those reported using approved mRNA-based vaccines (Comirnaty: 10^3^-10^4^ (89); mRNA-1273: 10^4^-10^5^ (90)), Ad-based vaccines (10^3^) (91) or subunit vaccines (10^3^-10^4^) (92). However, the protection achieved was not related with NAbs inhibiting the interaction of the virus with the host ACE2 receptor, since in our CoV2-BMEP candidate we exclude the receptor binding motif (RBM) of the S protein. Thus, other mechanisms such as inhibition of membrane fusion process, synergistic action of anti-S, anti-M and anti-N antibodies, Fc-mediated functions or induction of combined B and T cell immune responses might contribute to the protection observed. In terms of why two out of six mice were protected by vaccination, this phenomenon could be related to the nature of the immunogen, interaction with antigen-presenting cells and/or degree of intracellular processing. Nonetheless, the potent immunogenicity and efficacy triggered in some animals by the CoV2-BMEP protein highlights a novel vaccination approach distinct from the approved SARS-CoV-2 vaccines.

Recently, it was described the immunogenicity and anti-viral efficacy of a dendritic-cell targeting vaccine, named CD40.CoV2, that includes the RBD region (aa 318-541) in combination with three epitope enriched sequences from S1 (aa 125-250), S2 (aa 1056-1209) and N (aa 276-411) proteins of SARS-CoV-2 containing a large set of predicted CD4 and CD8 T and B cell conserved epitopes (93). Two doses of CD40.CoV2 vaccine administered by intraperitoneal (i.p.) route induced potent SARS-CoV-2-specific cross-reactive and NAbs correlated with anti-viral and protective activity against SARS-CoV-2 challenge in the hCD40/k18-hACE2 mouse model. The differences observed in term of efficacy between the CD40.CoV2 vaccine and our MVA-CoV2-BMEP candidate could be related with the immunization route (i.p. vs i.m.), with the virus challenge dose (2.5 × 10^4^ PFU vs 1 × 10^5^ PFU) or with the inclusion of the complete RBD region of the spike protein (absent in CoV2-BMEP).

Since the beginning of the COVID-19 pandemic, more than 100 citations in PubMed resource describe the design of multi-epitope vaccine candidates against SARS-CoV-2 using reverse vaccinology and immunoinformatic approaches including predicted B cell, CD4 and/or CD8 T cell epitopes from structural and non-structural viral proteins. However, most of these synthetic constructs have not been experimentally validated *in vivo* to define their capacity to elicit functional B and T cell responses and to confer protection against SARS-CoV-2 infection. In contrast, peptide-based vaccines using sequences from structural and non-structural proteins that contain protective B and/or T cell epitopes have been explored in clinic trials as alternative candidates to the approved SARS-CoV-2 vaccines. These include vaccines such as EpiVacCorona, CoVac-1, UB-612, CoVepiT or PepGNP-SARSCoV2.

The EpiVacCorona vaccine was produced by Rospotrebnadzor’s Vector Research Center and registered by the Russian Health Ministry on October 13, 2020, with no clinical effectiveness trials undergone. The vaccine contains three chemically synthesized short peptides from the SARS-CoV-2 spike protein conjugated to a chimeric carrier produced in *E. coli* (viral nucleocapsid protein fused to the bacterial maltose-binding protein (MBP) with a polyhistidine-tag) and adjuvanted with aluminum hydroxide. The preliminary evidence of safety and immunogenicity related to this vaccine was published in a local Russian journal (94), but the results of the phase III clinical trial have not been made public yet. However, in October 2022 Barchuk et al. reported the lack of efficacy of the EpiVacCorona vaccine against lung damage associated with COVID-19 during both Delta and Omicron surges (95).

CoVac-1 is a peptide-based vaccine containing six HLA-DR-restricted SARS-CoV-2 peptides from various viral proteins [spike, membrane, nucleocapsid, envelope and open reading frame 8 (ORF8)] combined with XS15, a Toll-like receptor 1/2 agonist, emulsified in Montanide ISA51 VG. In a phase I clinical study CoVac-1 demonstrated a favourable safety profile and elicited broad, potent and VoC-independent T cell responses (96). The vaccine has been also evaluated in patients with congenital or acquired B cell deficiencies with positive results (https://doi.org/10.21203/rs.3.rs-1693355/v1; preprint).

UB-612 is a protein/peptide subunit vaccine comprising S1-RBD-sFc protein and rationally designed promiscuous peptides corresponding to sarbecovirus conserved helper T cell and cytotoxic T lymphocyte epitopes of the membrane (M), nucleocapsid (N) and spike (S2) proteins. In phase I/II clinical trials the UB-612 vaccine was safe and well tolerated eliciting long-lasting NAb titers similar to levels detected in convalescent patients after two immunizations (97). Moreover, a third dose of vaccine induced potent S- and RBD-specific binding IgG and NAbs against multiple SARS-CoV-2 VoCs, including Omicron BA.1 and BA.2 sublineages (98). UB-612 is currently being tested in non-inferiority clinical trials as a heterologous booster for several authorized COVID-19 vaccines including ChAdOx nCoV-19 and BNT162b2.

CoVepiT is a multi-target, multi-variant vaccine created by OSE Immunotherapeutics Company based on 13 peptides from 11 SARS-CoV-2 structural and non-structural proteins targeting T cells. According to the company, CoVepiT demonstrated the ability to induce long-term CD8 T cell multi-epitope responses in preclinical and clinical trials (www.ose-immuno.com).

Finally, PepGNP-SARSCoV2, created by Emergex Vaccines Holding, comprises a specific cocktail of coronaviruses peptides targeting T cells mounted on a gold nanoparticle. The phase I study naNO-COVID (NCT05113862) evaluating the safety of 2 vaccine doses administered via an arm patch was completed in September 2022, but the results have not been published yet.

All these peptide-based vaccine candidates designed with the objective to provide cross-reactive B and/or T cell specific immune responses able to confer protection against the emerging virus variants represent alternative strategies to improve the immunogenicity and long-term efficacy against SARS-CoV-2.

In conclusion, this work provides a novel strategy distinct from the previously described approaches to advance in the development of vaccines based on the multi-patch sequence CoV2-BMEP containing conserved domains of structural proteins of SARS-CoV-2 that could confer a broader coverage of SARS-CoV-2-specific immune responses. The fact that both binding antibodies and specific T cell responses were directed against several proteins of SARS-CoV-2 represents an advantage over current vaccines that are only directed against the S component of the coronavirus. Moreover, we have achieved by vaccination full protection against mortality by SARS-CoV-2 in some vaccinated mice, indicative that this type of vaccine strategy is promising and it could be further improved. Indeed, due to the inability of current vaccines to generate sterile immunity to SARS-CoV-2 virus, novel vaccines should be considered to enhance and prolong the immune responses. Indeed, polyvalent CoV2-BMEP vaccine candidate could be used alone or in combination as a booster with other current vaccines to amplify and broaden the pre-existing B cell immune responses induced by infection or vaccination, establishing a long-term pool of memory cells able to control the SARS-CoV-2 and its VoCs, that are continuously emerging, and to prevent future outbreaks, especially in the high-risk population.

## Data availability statement

The original contributions presented in the study are included in the article/**Supplementary Material**. Further inquiries can be directed to the corresponding authors.

## Ethics statement

The Ethical Committee of Animal Experimentation (CEEA) of Centro Nacional de Biotecnología (CNB; Madrid, Spain) approved the animal experimental protocols, according to International European Union (EU) Guidelines Directive 2010/63/EU on protection of animals used for experimentation and other scientific purposes, Spanish National Law 32/2007 on animal welfare, exploitation, transport and sacrifice and Spanish National Royal Decree RD 53/2013 (permit number: PROEX 014/15). The experiments performed in k18-hACE2 humanized transgenic mice at CNB-CSIC and at the BSL-3 laboratory of Centro de Investigación en Sanidad Animal (CISA)-Instituto Nacional de Investigación y Tecnología Agraria y Alimentaria (INIA) (Madrid, Spain) were approved by the CEEA of the CNB-CSIC and CISA-INIA (Madrid, Spain) and by the Division of Animal Protection of the Comunidad de Madrid (PROEX 169.4/20). Animal procedures were conformed to International EU Guidelines Directive 2010/63/EU and Recommendation 2007/526/EC and to the Spanish law under the Royal Decree (RD 53/2013).

## Author contributions

Conceptualization: ME, CEG. Formal analysis: BP, LM-V, ML-B, COS, JRV, PS-C, CEG. Funding acquisition: ME, CEG. Investigation: BP, LM-V, CEG. Methodology: BP, LM-V, ML-B, CEG, JRV, PS-C, CZ, LS, EÁ, MR, MDV. Resources: ME, CEG. Supervision: ME, CEG. Validation: ME, CEG. Writing-original draft: CEG, BP, ME. Writing-review and editing: all authors. All authors contributed to the article and approved the submitted version.
