# Supplementary material for: Immunogenicity and efficacy of a novel multi-patch SARS-CoV-2/COVID-19 vaccine candidate
Source: Front Immunol. 2023 Jun 19;14:1160065. doi: 10.3389/fimmu.2023.1160065 (PMC10316789; doi:10.3389/fimmu.2023.1160065)
Supplement: Supplementary file 1 [file DataSheet_1.docx]

Supplementary Material

# Supplementary Figures and Tables

**Supplementary Table 1**

**Table S1**: Multi-patch segments included in the CoV2-BMEP synthetic protein.

| **Fragment** | **Peptide sequence** | **Start** | **End** | **Features** | **References** |
| --- | --- | --- | --- | --- | --- |
| S1 | YNSASFSTFKCYGVSPTKLNDLCF | 369 | 392 | Region targeted by cross-reactive NAbs (e.g. CR3022, S304, S2X35). Recognized by neutralizing antibodies from recovered SARS-CoV and SARS-CoV-2 patients. Includes immunoreactive CD8 T cell epitopes recognized by COVID-19 convalescent individuals, conserved in VoC. | (16, 17, 18, 21, 24, 25, 35, 37) |
| S2 | VNFNFNGLTGTGVLTESNKKF | 538 | 559 | Recognized by neutralizing antibodies from recovered SARS-CoV and SARS-CoV-2 patients. Related with favorable outcome (9,10,14–16). Includes immunoreactive CD8 T cell epitopes recognized by COVID-19 convalescent individuals. | (17, 18, 22, 28, 31, 34) |
| S3 | IPTNFTISVTTEILPVSMTKTSVDC | 714 | 738 | Includes immunodominant and neutralization lineal epitopes as well as overlapping CD4 and CD8 T cell epitopes recognized by COVID-19 convalescent patients. | (17, 24, 35) |
| S4 | DPSKPSKRSFIEDLLFNKVTLADAGFIK | 808 | 835 | Contains dominant and cross-reactive epitopes mediating long-term humoral immunity in COVID-19 convalescent individuals. Includes overlapping CD4 and CD8 T cell epitopes recognized by COVID-19 convalescent patients. Includes the conserved fusion peptide (FP) targeted by broadly neutralizing antibodies. | (10, 14, 17, 22, 27, 28, 31, 32, 38, 92) |
| S5 | IPFAMQMAYRFNGIGVTQNVLYENQKLIANQFN | 896 | 928 | Contains dominant and cross-reactive epitopes mediating long-term humoral immunity in COVID-19 convalescent individuals. Includes overlapping CD4 and CD8 T cell epitopes recognized by COVID-19 convalescent patients. | (17, 19, 27, 31, 32, 38) |
| S6 | VVNQNAQALNTLVKQLSSNFGAISS | 951 | 975 | Conserved region including overlapping CD8 T cell epitopes recognized by convalescent patients. Conserved in VoC. | (15, 17, 20, 32) |
| S7 | EIRASANLAATKMSECVLGQSKR | 1017 | 1039 | Conserved region including overlapping CD8 T cell epitopes recognized by convalescent patients. Conserved in VoC. | (15, 17, 20, 32) |
| S8 | LNEVAKNLNESLIDLQELGKYE | 1186 | 1207 | Contains cross-reactive B and T cell epitopes recognized by convalescent patients. Conserved in VoC. | (15, 17, 18, 20, 29, 32, 35) |
| M1 | YFIASFRLFARTRSMWSFNPETN | 95 | 117 | Contains cross-reactive B and T cell epitopes recognized by convalescent patients. | (17, 30, 31, 32) |
| N1 | ASWFTALTQHGKEDLKFPRGQGVPIN | 50 | 75 | Contains cross-reactive B and T cell epitopes recognized by convalescent patients. | (17, 24, 25, 32, 34, 36) |
| N2 | NNAAIVLQLPQGTTLPKGFYAEGS | 153 | 176 | Recognized by neutralizing antibodies from recovered SARS-CoV and SARS-CoV-2 patients. Includes immunoreactive CD8 T cell epitopes recognized by COVID-19 convalescent individuals. | (17, 18, 19, 24, 26, 32, 33, 35) |

**Supplementary Table 2**

Predicted coverage for MHC class I epitopes for CoV2-BMEP

| Population / area | MHC class I | | |
| --- | --- | --- | --- |
|  | coveragea | average_hitb | pc90c |
| Algeria | 98.8% | 17.47 | 9.35 |
| Algeria Arab | 98.8% | 17.47 | 9.35 |
| Argentina | 99.46% | 18.4 | 10.49 |
| Argentina Amerindian | 99.96% | 18.59 | 10.73 |
| Argentina Caucasoid | 97.6% | 17.05 | 7.98 |
| Australia | 99.99% | 18.44 | 10.76 |
| Australia Australian Aborigines | 99.99% | 18.44 | 10.76 |
| Austria | 98.1% | 17.76 | 7.99 |
| Austria Caucasoid | 98.1% | 17.76 | 7.99 |
| Belarus | 45.3% | 4.02 | 1.28 |
| Belarus Caucasoid | 45.3% | 4.02 | 1.28 |
| Belgium | 92.49% | 14.19 | 7.03 |
| Belgium Caucasoid | 92.49% | 14.19 | 7.03 |
| Bolivia | 99.98% | 17.1 | 9.79 |
| Bolivia Amerindian | 99.98% | 17.1 | 9.79 |
| Borneo | 99.94% | 18.09 | 9.56 |
| Borneo Austronesian | 99.94% | 18.09 | 9.56 |
| Brazil | 99.79% | 18.78 | 11.73 |
| Brazil Amerindian | 100.0% | 18.7 | 12.16 |
| Brazil Caucasoid | 98.75% | 18.44 | 11.01 |
| Brazil Mixed | 98.81% | 17.87 | 9.25 |
| Brazil Mulatto | 97.01% | 17.17 | 9.06 |
| Bulgaria | 98.39% | 19.05 | 9.67 |
| Bulgaria Caucasoid | 98.39% | 19.05 | 9.67 |
| Cameroon | 95.38% | 15.52 | 7.64 |
| Cameroon Black | 95.38% | 15.52 | 7.64 |
| Canada | 97.95% | 14.51 | 9.08 |
| Canada Amerindian | 97.95% | 14.51 | 9.08 |
| Cape Verde | 95.23% | 15.53 | 7.35 |
| Cape Verde Black | 95.23% | 15.53 | 7.35 |
| Central Africa | 94.62% | 15.79 | 7.41 |
| Central African Republic | 94.98% | 14.38 | 7.32 |
| Central African Republic Black | 94.98% | 14.38 | 7.32 |
| Central America | 99.83% | 16.97 | 9.5 |
| Chile | 96.24% | 15.24 | 7.85 |
| Chile Amerindian | 97.28% | 13.36 | 8.14 |
| Chile Mixed | 85.79% | 12.28 | 4.22 |
| China | 99.67% | 18.63 | 11.41 |
| China Oriental | 99.67% | 18.63 | 11.41 |
| Colombia | 99.5% | 18.03 | 10.23 |
| Colombia Amerindian | 99.83% | 18.02 | 10.62 |
| Colombia Black | 98.28% | 17.04 | 8.02 |
| Colombia Mestizo | 91.59% | 14.6 | 6.55 |
| Congo | 82.6% | 11.76 | 2.87 |
| Congo Black | 82.6% | 11.76 | 2.87 |
| Cook Islands | 99.97% | 16.82 | 9.56 |
| Cook Islands Polynesian | 99.97% | 16.82 | 9.56 |
| Costa Rica | 99.84% | 17.51 | 9.89 |
| Costa Rica Mestizo | 99.84% | 17.51 | 9.89 |
| Croatia | 96.48% | 18.09 | 8.78 |
| Croatia Caucasoid | 96.48% | 18.09 | 8.78 |
| Cuba | 98.23% | 17.71 | 11.04 |
| Cuba Mixed | 98.23% | 17.71 | 11.04 |
| Czech Republic | 98.26% | 17.79 | 9.41 |
| Czech Republic Caucasoid | 98.32% | 18.03 | 9.49 |
| Czech Republic Other | 97.16% | 15.36 | 9.03 |
| Denmark | 91.71% | 14.1 | 7.11 |
| Denmark Caucasoid | 91.71% | 14.1 | 7.11 |
| East Africa | 97.34% | 16.55 | 8.98 |
| East Asia | 99.43% | 18.44 | 10.85 |
| Ecuador | 100.0% | 18.36 | 10.18 |
| Ecuador Amerindian | 100.0% | 18.36 | 10.18 |
| England | 99.2% | 17.56 | 7.99 |
| England Caucasoid | 99.2% | 17.56 | 7.99 |
| Equatorial Guinea | 82.61% | 12.59 | 2.88 |
| Equatorial Guinea Black | 82.61% | 12.59 | 2.88 |
| Ethiopia | 95.12% | 15.5 | 7.68 |
| Ethiopia Black | 95.12% | 15.5 | 7.68 |
| Europe | 98.8% | 18.21 | 9.55 |
| Fiji | 99.99% | 17.52 | 9.71 |
| Fiji Melanesian | 99.99% | 17.52 | 9.71 |
| Finland | 62.05% | 7.71 | 1.58 |
| Finland Caucasoid | 62.05% | 7.71 | 1.58 |
| France | 98.38% | 17.31 | 7.78 |
| France Caucasoid | 98.38% | 17.31 | 7.78 |
| Gabon | 55.91% | 6.82 | 2.04 |
| Gabon Black | 55.91% | 6.82 | 2.04 |
| Georgia | 97.55% | 16.75 | 9.07 |
| Georgia Caucasoid | 97.55% | 16.75 | 9.07 |
| Germany | 98.29% | 17.69 | 7.95 |
| Germany Caucasoid | 98.29% | 17.69 | 7.95 |
| Greece | 99.33% | 20.02 | 11.65 |
| Greece Caucasoid | 99.33% | 20.02 | 11.65 |
| Guatemala | 98.31% | 14.36 | 9.16 |
| Guatemala Amerindian | 98.31% | 14.36 | 9.16 |
| Guinea-Bissau | 90.76% | 14.17 | 7.15 |
| Guinea-Bissau Black | 90.76% | 14.17 | 7.15 |
| India | 98.94% | 18.32 | 10.99 |
| India Asian | 98.94% | 18.32 | 10.99 |
| Indonesia | 99.96% | 17.93 | 9.7 |
| Indonesia Austronesian | 99.96% | 17.93 | 9.7 |
| Iran | 99.05% | 16.77 | 7.87 |
| Iran Kurd | 99.19% | 16.26 | 7.79 |
| Iran Persian | 98.96% | 16.75 | 7.86 |
| Ireland Northern | 99.34% | 17.72 | 7.98 |
| Ireland Northern Caucasoid | 99.34% | 17.72 | 7.98 |
| Ireland South | 99.06% | 16.92 | 7.74 |
| Ireland South Caucasoid | 99.06% | 16.92 | 7.74 |
| Israel | 97.84% | 16.9 | 8.53 |
| Israel Arab | 98.36% | 16.93 | 8.78 |
| Israel Jew | 97.61% | 16.85 | 8.24 |
| Italy | 99.33% | 15.31 | 7.44 |
| Italy Caucasoid | 99.33% | 15.31 | 7.44 |
| Jamaica | 76.28% | 9.38 | 2.11 |
| Jamaica Black | 76.28% | 9.38 | 2.11 |
| Japan | 99.65% | 18.99 | 11.26 |
| Japan Oriental | 99.65% | 18.99 | 11.26 |
| Jordan | 99.48% | 13.58 | 6.53 |
| Jordan Arab | 99.48% | 13.58 | 6.53 |
| Kiribati | 100.0% | 16.92 | 9.41 |
| Kiribati Micronesian | 100.0% | 16.92 | 9.41 |
| Korea; South | 98.84% | 17.72 | 9.85 |
| Korea; South Oriental | 98.84% | 17.72 | 9.85 |
| Lebanon | 99.3% | 18.21 | 9.67 |
| Lebanon Arab | 99.3% | 18.21 | 9.67 |
| Macedonia | 99.04% | 19.89 | 11.46 |
| Macedonia Caucasoid | 99.04% | 19.89 | 11.46 |
| Malaysia | 99.87% | 19.08 | 11.62 |
| Malaysia Austronesian | 99.95% | 18.82 | 11.46 |
| Malaysia Oriental | 99.45% | 18.59 | 11.37 |
| Martinique | 97.28% | 16.92 | 7.94 |
| Martinique Black | 97.28% | 16.92 | 7.94 |
| Mexico | 99.85% | 18.31 | 10.6 |
| Mexico Amerindian | 99.97% | 18.55 | 10.87 |
| Mexico Mestizo | 99.67% | 18.04 | 9.95 |
| Mongolia | 99.71% | 18.47 | 11.51 |
| Mongolia Oriental | 99.71% | 18.47 | 11.51 |
| Morocco | 97.23% | 15.69 | 7.63 |
| Morocco Arab | 96.59% | 15.19 | 7.5 |
| Morocco Caucasoid | 98.04% | 16.52 | 7.97 |
| Nauru | 99.98% | 17.4 | 9.84 |
| Nauru Micronesian | 99.98% | 17.4 | 9.84 |
| Netherlands | 96.41% | 16.12 | 7.44 |
| Netherlands Caucasoid | 96.41% | 16.12 | 7.44 |
| New Caledonia | 100.0% | 18.21 | 9.81 |
| New Caledonia Melanesian | 100.0% | 18.21 | 9.81 |
| New Zealand | 99.91% | 16.98 | 9.56 |
| New Zealand Polynesian | 99.91% | 16.98 | 9.56 |
| Niue | 99.98% | 15.84 | 8.02 |
| Niue Polynesian | 99.98% | 15.84 | 8.02 |
| North Africa | 98.08% | 16.88 | 8.0 |
| North America | 98.7% | 17.71 | 9.07 |
| Northeast Asia | 99.67% | 18.63 | 11.41 |
| Norway | 98.31% | 16.77 | 7.6 |
| Norway Caucasoid | 98.31% | 16.77 | 7.6 |
| Oceania | 99.98% | 19.28 | 15.75 |
| Pakistan | 7.05% | 0.92 | 1.4 |
| Pakistan Asian | 6.68% | 0.87 | 1.39 |
| Pakistan Mixed | 7.84% | 1.02 | 1.41 |
| Papua New Guinea | 100.0% | 19.63 | 15.26 |
| Papua New Guinea Melanesian | 100.0% | 19.63 | 15.26 |
| Paraguay | 100.0% | 16.56 | 10.57 |
| Paraguay Amerindian | 100.0% | 16.56 | 10.57 |
| Peru | 99.6% | 17.3 | 9.7 |
| Peru Amerindian | 99.6% | 17.3 | 9.7 |
| Philippines | 100.0% | 17.52 | 11.15 |
| Philippines Austronesian | 100.0% | 17.52 | 11.15 |
| Poland | 98.4% | 18.46 | 9.38 |
| Poland Caucasoid | 98.4% | 18.46 | 9.38 |
| Portugal | 97.82% | 17.22 | 8.18 |
| Portugal Caucasoid | 97.82% | 17.22 | 8.18 |
| Russia | 99.48% | 18.68 | 11.09 |
| Russia Caucasoid | 98.92% | 18.7 | 10.22 |
| Russia Other | 99.83% | 19.68 | 11.82 |
| Russia Siberian | 99.49% | 18.2 | 10.75 |
| Rwanda | 93.08% | 14.99 | 7.09 |
| Rwanda Black | 93.08% | 14.99 | 7.09 |
| Samoa | 99.93% | 17.57 | 11.14 |
| Samoa Polynesian | 99.93% | 17.57 | 11.14 |
| Sao Tome and Principe | 97.62% | 16.63 | 7.59 |
| Sao Tome and Principe Black | 97.62% | 16.63 | 7.59 |
| Saudi Arabia | 96.58% | 15.73 | 7.84 |
| Saudi Arabia Arab | 96.58% | 15.73 | 7.84 |
| Scotland | 96.39% | 15.49 | 7.39 |
| Scotland Caucasoid | 96.39% | 15.49 | 7.39 |
| Senegal | 81.94% | 12.59 | 2.77 |
| Senegal Black | 81.94% | 12.59 | 2.77 |
| Singapore | 99.9% | 17.92 | 9.67 |
| Singapore Austronesian | 99.9% | 17.92 | 9.67 |
| Slovakia | 22.38% | 1.87 | 0.9 |
| Slovakia Caucasoid | 22.38% | 1.87 | 0.9 |
| Slovenia | 98.65% | 18.76 | 9.65 |
| Slovenia Caucasoid | 98.65% | 18.76 | 9.65 |
| South Africa | 53.35% | 3.84 | 1.07 |
| South Africa Black | 53.35% | 3.84 | 1.07 |
| South America | 99.74% | 18.67 | 11.1 |
| South Asia | 99.01% | 18.35 | 11.02 |
| Southeast Asia | 99.9% | 18.8 | 11.94 |
| Southwest Asia | 99.15% | 15.18 | 6.83 |
| Spain | 98.45% | 17.69 | 9.44 |
| Spain Caucasoid | 98.45% | 17.69 | 9.43 |
| Spain Jew | 0.0% | 0.0 | 1.4 |
| Spain Other | 25.61% | 2.95 | 1.48 |
| Sudan | 95.16% | 16.08 | 7.52 |
| Sudan Mixed | 95.16% | 16.08 | 7.52 |
| Sweden | 98.83% | 18.82 | 9.7 |
| Sweden Caucasoid | 98.83% | 18.82 | 9.7 |
| Taiwan | 99.98% | 18.51 | 13.06 |
| Taiwan Oriental | 99.98% | 18.51 | 13.06 |
| Thailand | 99.88% | 19.01 | 11.65 |
| Thailand Oriental | 99.88% | 19.01 | 11.65 |
| Tokelau | 99.91% | 14.95 | 8.22 |
| Tokelau Polynesian | 99.91% | 14.95 | 8.22 |
| Tonga | 100.0% | 18.15 | 11.51 |
| Tonga Polynesian | 100.0% | 18.15 | 11.51 |
| Tunisia | 98.07% | 16.54 | 7.92 |
| Tunisia Arab | 98.07% | 16.15 | 7.82 |
| Tunisia Berber | 97.08% | 17.23 | 8.87 |
| Turkey | 98.46% | 17.75 | 9.29 |
| Turkey Caucasoid | 98.46% | 17.75 | 9.29 |
| Ukraine | 50.64% | 4.51 | 1.42 |
| Ukraine Caucasoid | 50.64% | 4.51 | 1.42 |
| United Arab Emirates | 34.71% | 2.41 | 0.77 |
| United Arab Emirates Arab | 34.71% | 2.41 | 0.77 |
| United States | 98.61% | 17.65 | 8.23 |
| United States Amerindian | 99.99% | 17.6 | 10.59 |
| United States Asian | 99.61% | 18.54 | 11.29 |
| United States Austronesian | 99.96% | 17.95 | 11.31 |
| United States Black | 96.91% | 16.54 | 7.73 |
| United States Caucasoid | 98.08% | 17.15 | 7.75 |
| United States Hispanic | 98.99% | 17.63 | 9.7 |
| United States Mestizo | 99.29% | 17.96 | 9.94 |
| United States Polynesian | 99.95% | 18.33 | 11.82 |
| Venezuela | 100.0% | 16.87 | 10.51 |
| Venezuela Amerindian | 100.0% | 16.4 | 10.46 |
| Venezuela Mixed | 10.13% | 1.14 | 0.78 |
| Vietnam | 99.85% | 18.14 | 9.9 |
| Vietnam Oriental | 99.85% | 18.14 | 9.9 |
| Wales | 4.35% | 0.61 | 1.46 |
| Wales Caucasoid | 4.35% | 0.61 | 1.46 |
| West Africa | 92.93% | 15.23 | 7.13 |
| West Indies | 98.29% | 17.32 | 9.3 |
| World | 99.09% | 18.24 | 10.07 |
| Zimbabwe | 97.34% | 16.55 | 8.98 |
| Zimbabwe Black | 97.34% | 16.55 | 8.98 |
| Average | 91.59 | 15.7 | 8.38 |
| Standard deviation | 20.44 | 4.43 | 3.02 |

a projected population coverage; b average number of epitope hits / HLA combinations recognized by the population; c minimum number of epitope hits / HLA combinations recognized by 90% of the population.

**Supplementary Table 3**

Predicted population coverage of MHC class II epitopes for CoV2-BMEP

| Population / area | MHC class II | | |
| --- | --- | --- | --- |
|  | coveragea | average_hitb | pc90c |
| Algeria | 98.8% | 17.47 | 9.35 |
| Algeria Arab | 98.8% | 17.47 | 9.35 |
| Argentina | 99.46% | 18.4 | 10.49 |
| Argentina Amerindian | 99.96% | 18.59 | 10.73 |
| Argentina Caucasoid | 97.6% | 17.05 | 7.98 |
| Australia | 99.99% | 18.44 | 10.76 |
| Australia Australian Aborigines | 99.99% | 18.44 | 10.76 |
| Austria | 98.1% | 17.76 | 7.99 |
| Austria Caucasoid | 98.1% | 17.76 | 7.99 |
| Belarus | 45.3% | 4.02 | 1.28 |
| Belarus Caucasoid | 45.3% | 4.02 | 1.28 |
| Belgium | 92.49% | 14.19 | 7.03 |
| Belgium Caucasoid | 92.49% | 14.19 | 7.03 |
| Bolivia | 99.98% | 17.1 | 9.79 |
| Bolivia Amerindian | 99.98% | 17.1 | 9.79 |
| Borneo | 99.94% | 18.09 | 9.56 |
| Borneo Austronesian | 99.94% | 18.09 | 9.56 |
| Brazil | 99.79% | 18.78 | 11.73 |
| Brazil Amerindian | 100.0% | 18.7 | 12.16 |
| Brazil Caucasoid | 98.75% | 18.44 | 11.01 |
| Brazil Mixed | 98.81% | 17.87 | 9.25 |
| Brazil Mulatto | 97.01% | 17.17 | 9.06 |
| Bulgaria | 98.39% | 19.05 | 9.67 |
| Bulgaria Caucasoid | 98.39% | 19.05 | 9.67 |
| Cameroon | 95.38% | 15.52 | 7.64 |
| Cameroon Black | 95.38% | 15.52 | 7.64 |
| Canada | 97.95% | 14.51 | 9.08 |
| Canada Amerindian | 97.95% | 14.51 | 9.08 |
| Cape Verde | 95.23% | 15.53 | 7.35 |
| Cape Verde Black | 95.23% | 15.53 | 7.35 |
| Central Africa | 94.62% | 15.79 | 7.41 |
| Central African Republic | 94.98% | 14.38 | 7.32 |
| Central African Republic Black | 94.98% | 14.38 | 7.32 |
| Central America | 99.83% | 16.97 | 9.5 |
| Chile | 96.24% | 15.24 | 7.85 |
| Chile Amerindian | 97.28% | 13.36 | 8.14 |
| Chile Mixed | 85.79% | 12.28 | 4.22 |
| China | 99.67% | 18.63 | 11.41 |
| China Oriental | 99.67% | 18.63 | 11.41 |
| Colombia | 99.5% | 18.03 | 10.23 |
| Colombia Amerindian | 99.83% | 18.02 | 10.62 |
| Colombia Black | 98.28% | 17.04 | 8.02 |
| Colombia Mestizo | 91.59% | 14.6 | 6.55 |
| Congo | 82.6% | 11.76 | 2.87 |
| Congo Black | 82.6% | 11.76 | 2.87 |
| Cook Islands | 99.97% | 16.82 | 9.56 |
| Cook Islands Polynesian | 99.97% | 16.82 | 9.56 |
| Costa Rica | 99.84% | 17.51 | 9.89 |
| Costa Rica Mestizo | 99.84% | 17.51 | 9.89 |
| Croatia | 96.48% | 18.09 | 8.78 |
| Croatia Caucasoid | 96.48% | 18.09 | 8.78 |
| Cuba | 98.23% | 17.71 | 11.04 |
| Cuba Mixed | 98.23% | 17.71 | 11.04 |
| Czech Republic | 98.26% | 17.79 | 9.41 |
| Czech Republic Caucasoid | 98.32% | 18.03 | 9.49 |
| Czech Republic Other | 97.16% | 15.36 | 9.03 |
| Denmark | 91.71% | 14.1 | 7.11 |
| Denmark Caucasoid | 91.71% | 14.1 | 7.11 |
| East Africa | 97.34% | 16.55 | 8.98 |
| East Asia | 99.43% | 18.44 | 10.85 |
| Ecuador | 100.0% | 18.36 | 10.18 |
| Ecuador Amerindian | 100.0% | 18.36 | 10.18 |
| England | 99.2% | 17.56 | 7.99 |
| England Caucasoid | 99.2% | 17.56 | 7.99 |
| Equatorial Guinea | 82.61% | 12.59 | 2.88 |
| Equatorial Guinea Black | 82.61% | 12.59 | 2.88 |
| Ethiopia | 95.12% | 15.5 | 7.68 |
| Ethiopia Black | 95.12% | 15.5 | 7.68 |
| Europe | 98.8% | 18.21 | 9.55 |
| Fiji | 99.99% | 17.52 | 9.71 |
| Fiji Melanesian | 99.99% | 17.52 | 9.71 |
| Finland | 62.05% | 7.71 | 1.58 |
| Finland Caucasoid | 62.05% | 7.71 | 1.58 |
| France | 98.38% | 17.31 | 7.78 |
| France Caucasoid | 98.38% | 17.31 | 7.78 |
| Gabon | 55.91% | 6.82 | 2.04 |
| Gabon Black | 55.91% | 6.82 | 2.04 |
| Georgia | 97.55% | 16.75 | 9.07 |
| Georgia Caucasoid | 97.55% | 16.75 | 9.07 |
| Germany | 98.29% | 17.69 | 7.95 |
| Germany Caucasoid | 98.29% | 17.69 | 7.95 |
| Greece | 99.33% | 20.02 | 11.65 |
| Greece Caucasoid | 99.33% | 20.02 | 11.65 |
| Guatemala | 98.31% | 14.36 | 9.16 |
| Guatemala Amerindian | 98.31% | 14.36 | 9.16 |
| Guinea-Bissau | 90.76% | 14.17 | 7.15 |
| Guinea-Bissau Black | 90.76% | 14.17 | 7.15 |
| India | 98.94% | 18.32 | 10.99 |
| India Asian | 98.94% | 18.32 | 10.99 |
| Indonesia | 99.96% | 17.93 | 9.7 |
| Indonesia Austronesian | 99.96% | 17.93 | 9.7 |
| Iran | 99.05% | 16.77 | 7.87 |
| Iran Kurd | 99.19% | 16.26 | 7.79 |
| Iran Persian | 98.96% | 16.75 | 7.86 |
| Ireland Northern | 99.34% | 17.72 | 7.98 |
| Ireland Northern Caucasoid | 99.34% | 17.72 | 7.98 |
| Ireland South | 99.06% | 16.92 | 7.74 |
| Ireland South Caucasoid | 99.06% | 16.92 | 7.74 |
| Israel | 97.84% | 16.9 | 8.53 |
| Israel Arab | 98.36% | 16.93 | 8.78 |
| Israel Jew | 97.61% | 16.85 | 8.24 |
| Italy | 99.33% | 15.31 | 7.44 |
| Italy Caucasoid | 99.33% | 15.31 | 7.44 |
| Jamaica | 76.28% | 9.38 | 2.11 |
| Jamaica Black | 76.28% | 9.38 | 2.11 |
| Japan | 99.65% | 18.99 | 11.26 |
| Japan Oriental | 99.65% | 18.99 | 11.26 |
| Jordan | 99.48% | 13.58 | 6.53 |
| Jordan Arab | 99.48% | 13.58 | 6.53 |
| Kiribati | 100.0% | 16.92 | 9.41 |
| Kiribati Micronesian | 100.0% | 16.92 | 9.41 |
| Korea; South | 98.84% | 17.72 | 9.85 |
| Korea; South Oriental | 98.84% | 17.72 | 9.85 |
| Lebanon | 99.3% | 18.21 | 9.67 |
| Lebanon Arab | 99.3% | 18.21 | 9.67 |
| Macedonia | 99.04% | 19.89 | 11.46 |
| Macedonia Caucasoid | 99.04% | 19.89 | 11.46 |
| Malaysia | 99.87% | 19.08 | 11.62 |
| Malaysia Austronesian | 99.95% | 18.82 | 11.46 |
| Malaysia Oriental | 99.45% | 18.59 | 11.37 |
| Martinique | 97.28% | 16.92 | 7.94 |
| Martinique Black | 97.28% | 16.92 | 7.94 |
| Mexico | 99.85% | 18.31 | 10.6 |
| Mexico Amerindian | 99.97% | 18.55 | 10.87 |
| Mexico Mestizo | 99.67% | 18.04 | 9.95 |
| Mongolia | 99.71% | 18.47 | 11.51 |
| Mongolia Oriental | 99.71% | 18.47 | 11.51 |
| Morocco | 97.23% | 15.69 | 7.63 |
| Morocco Arab | 96.59% | 15.19 | 7.5 |
| Morocco Caucasoid | 98.04% | 16.52 | 7.97 |
| Nauru | 99.98% | 17.4 | 9.84 |
| Nauru Micronesian | 99.98% | 17.4 | 9.84 |
| Netherlands | 96.41% | 16.12 | 7.44 |
| Netherlands Caucasoid | 96.41% | 16.12 | 7.44 |
| New Caledonia | 100.0% | 18.21 | 9.81 |
| New Caledonia Melanesian | 100.0% | 18.21 | 9.81 |
| New Zealand | 99.91% | 16.98 | 9.56 |
| New Zealand Polynesian | 99.91% | 16.98 | 9.56 |
| Niue | 99.98% | 15.84 | 8.02 |
| Niue Polynesian | 99.98% | 15.84 | 8.02 |
| North Africa | 98.08% | 16.88 | 8.0 |
| North America | 98.7% | 17.71 | 9.07 |
| Northeast Asia | 99.67% | 18.63 | 11.41 |
| Norway | 98.31% | 16.77 | 7.6 |
| Norway Caucasoid | 98.31% | 16.77 | 7.6 |
| Oceania | 99.98% | 19.28 | 15.75 |
| Pakistan | 7.05% | 0.92 | 1.4 |
| Pakistan Asian | 6.68% | 0.87 | 1.39 |
| Pakistan Mixed | 7.84% | 1.02 | 1.41 |
| Papua New Guinea | 100.0% | 19.63 | 15.26 |
| Papua New Guinea Melanesian | 100.0% | 19.63 | 15.26 |
| Paraguay | 100.0% | 16.56 | 10.57 |
| Paraguay Amerindian | 100.0% | 16.56 | 10.57 |
| Peru | 99.6% | 17.3 | 9.7 |
| Peru Amerindian | 99.6% | 17.3 | 9.7 |
| Philippines | 100.0% | 17.52 | 11.15 |
| Philippines Austronesian | 100.0% | 17.52 | 11.15 |
| Poland | 98.4% | 18.46 | 9.38 |
| Poland Caucasoid | 98.4% | 18.46 | 9.38 |
| Portugal | 97.82% | 17.22 | 8.18 |
| Portugal Caucasoid | 97.82% | 17.22 | 8.18 |
| Russia | 99.48% | 18.68 | 11.09 |
| Russia Caucasoid | 98.92% | 18.7 | 10.22 |
| Russia Other | 99.83% | 19.68 | 11.82 |
| Russia Siberian | 99.49% | 18.2 | 10.75 |
| Rwanda | 93.08% | 14.99 | 7.09 |
| Rwanda Black | 93.08% | 14.99 | 7.09 |
| Samoa | 99.93% | 17.57 | 11.14 |
| Samoa Polynesian | 99.93% | 17.57 | 11.14 |
| Sao Tome and Principe | 97.62% | 16.63 | 7.59 |
| Sao Tome and Principe Black | 97.62% | 16.63 | 7.59 |
| Saudi Arabia | 96.58% | 15.73 | 7.84 |
| Saudi Arabia Arab | 96.58% | 15.73 | 7.84 |
| Scotland | 96.39% | 15.49 | 7.39 |
| Scotland Caucasoid | 96.39% | 15.49 | 7.39 |
| Senegal | 81.94% | 12.59 | 2.77 |
| Senegal Black | 81.94% | 12.59 | 2.77 |
| Singapore | 99.9% | 17.92 | 9.67 |
| Singapore Austronesian | 99.9% | 17.92 | 9.67 |
| Slovakia | 22.38% | 1.87 | 0.9 |
| Slovakia Caucasoid | 22.38% | 1.87 | 0.9 |
| Slovenia | 98.65% | 18.76 | 9.65 |
| Slovenia Caucasoid | 98.65% | 18.76 | 9.65 |
| South Africa | 53.35% | 3.84 | 1.07 |
| South Africa Black | 53.35% | 3.84 | 1.07 |
| South America | 99.74% | 18.67 | 11.1 |
| South Asia | 99.01% | 18.35 | 11.02 |
| Southeast Asia | 99.9% | 18.8 | 11.94 |
| Southwest Asia | 99.15% | 15.18 | 6.83 |
| Spain | 98.45% | 17.69 | 9.44 |
| Spain Caucasoid | 98.45% | 17.69 | 9.43 |
| Spain Jew | 0.0% | 0.0 | 1.4 |
| Spain Other | 25.61% | 2.95 | 1.48 |
| Sudan | 95.16% | 16.08 | 7.52 |
| Sudan Mixed | 95.16% | 16.08 | 7.52 |
| Sweden | 98.83% | 18.82 | 9.7 |
| Sweden Caucasoid | 98.83% | 18.82 | 9.7 |
| Taiwan | 99.98% | 18.51 | 13.06 |
| Taiwan Oriental | 99.98% | 18.51 | 13.06 |
| Thailand | 99.88% | 19.01 | 11.65 |
| Thailand Oriental | 99.88% | 19.01 | 11.65 |
| Tokelau | 99.91% | 14.95 | 8.22 |
| Tokelau Polynesian | 99.91% | 14.95 | 8.22 |
| Tonga | 100.0% | 18.15 | 11.51 |
| Tonga Polynesian | 100.0% | 18.15 | 11.51 |
| Tunisia | 98.07% | 16.54 | 7.92 |
| Tunisia Arab | 98.07% | 16.15 | 7.82 |
| Tunisia Berber | 97.08% | 17.23 | 8.87 |
| Turkey | 98.46% | 17.75 | 9.29 |
| Turkey Caucasoid | 98.46% | 17.75 | 9.29 |
| Ukraine | 50.64% | 4.51 | 1.42 |
| Ukraine Caucasoid | 50.64% | 4.51 | 1.42 |
| United Arab Emirates | 34.71% | 2.41 | 0.77 |
| United Arab Emirates Arab | 34.71% | 2.41 | 0.77 |
| United States | 98.61% | 17.65 | 8.23 |
| United States Amerindian | 99.99% | 17.6 | 10.59 |
| United States Asian | 99.61% | 18.54 | 11.29 |
| United States Austronesian | 99.96% | 17.95 | 11.31 |
| United States Black | 96.91% | 16.54 | 7.73 |
| United States Caucasoid | 98.08% | 17.15 | 7.75 |
| United States Hispanic | 98.99% | 17.63 | 9.7 |
| United States Mestizo | 99.29% | 17.96 | 9.94 |
| United States Polynesian | 99.95% | 18.33 | 11.82 |
| Venezuela | 100.0% | 16.87 | 10.51 |
| Venezuela Amerindian | 100.0% | 16.4 | 10.46 |
| Venezuela Mixed | 10.13% | 1.14 | 0.78 |
| Vietnam | 99.85% | 18.14 | 9.9 |
| Vietnam Oriental | 99.85% | 18.14 | 9.9 |
| Wales | 4.35% | 0.61 | 1.46 |
| Wales Caucasoid | 4.35% | 0.61 | 1.46 |
| West Africa | 92.93% | 15.23 | 7.13 |
| West Indies | 98.29% | 17.32 | 9.3 |
| World | 99.09% | 18.24 | 10.07 |
| Zimbabwe | 97.34% | 16.55 | 8.98 |
| Zimbabwe Black | 97.34% | 16.55 | 8.98 |
| Average | 91.59 | 15.7 | 8.38 |
| Standard deviation | 20.44 | 4.43 | 3.02 |

a projected population coverage; b average number of epitope hits / HLA combinations recognized by the population; c minimum number of epitope hits / HLA combinations recognized by 90% of the population.

**Supplementary Figure 1.** Gating strategies used for the identification of the different immune cell populations in muscle (A) and DLNs (B) by flow cytometry.

**Supplementary Figure 2.** IgA (left) and IgM (right) binding antibody levels to SARS-CoV-2 S protein from Wuhan reference strain elicited in serum from immunized individual mice at 20 and 42 days post-boost measured as OD_450_ at a serum dilution of 1:50 by ELISA. Data are shown as forms for each animal with mean and SD. *, p < 0.05; **, p < 0.005.
